# Ethanol-Guided Hybridization of Extracellular Vesicles with Liquid-Crystalline Lipid Nanoparticles

**DOI:** 10.1021/acsami.5c22972

**Published:** 2026-01-26

**Authors:** Valentina Pacciani, Jacopo Cardellini, Arianna Balestri, Marta Rojas-Rodríguez, Martino Calamai, Mattia Tiboni, Luca Casettari, Catherine E. Saunders, Anam A. Karimi, Gennaro Sanità, Emanuela Esposito, Andrea Zendrini, Annalisa Radeghieri, Lucia Paolini, Paolo Bergese, Costanza Montis, Lucrezia Caselli, Debora Berti

**Affiliations:** 1 Department of Chemistry “Ugo Schiff”, 201779 University of Florence, Sesto Fiorentino, 50019 Florence, Italy; 2 CSGI, Center for Colloid and Surface Science, Sesto Fiorentino, 50019 Florence, Italy; 3 European Laboratory for Non-Linear Spectroscopy, Via Nello Carrara 1, 50019 Sesto Fiorentino, Italy; 4 National Institute of Optics, National Research Council (CNR-INO), 50125 Sesto Fiorentino, Italy; 5 Department of Biomolecular Sciences, University of Urbino Carlo Bo, Via Ca’ le Suore, 2, 61029 Urbino, Italy; 6 SPARTA Biodiscovery, Scale Space White City, 58 Wood Lane, London W12 7RZ, United Kingdom; 7 EYE-Lab, Institute of Applied Sciences and Intelligent Systems, National Research Council, Via Pietro Castellino 111, 80131 Napoli, Italy; 8 Department of Molecular and Translational Medicine, University of Brescia, 25123 Brescia, Italy; 9 Department of Medical and Surgical Specialties, Radiological Sciences and Public Health, 9297 University of Brescia, 25123 Brescia, Italy

**Keywords:** microfluidic engineering, extracellular vesicles, hybrid lipid nanoparticles, cubosomes, red blood cell EVs

## Abstract

Hybrid nanosystems that integrate biological and synthetic lipid assemblies hold great promise for tailoring nanoscale interfaces with programmable chemical and structural functionality. However, existing approaches to hybridize extracellular vesicles (EVs) with lipid nanoparticles (LNPs) compromise either the EV bioactivity or the native supramolecular organization of synthetic LNPs, undermining structure-dependent functionality. Here, we introduce an ethanol-mediated microfluidic assembly route that enables the *in situ* formation and hybridization of nonlamellar liquid-crystalline lipid nanoparticles (LCNPs) with red-blood-cell-derived EVs (RBCEVs) in a single step. This process exploits ethanol-induced interfacial reorganization to drive EV incorporation without compromising the LCNP cubic architecture. Synchrotron small-angle X-ray scattering (SAXS) and cryogenic electron microscopy reveal hybrid nanoparticles that retain long-range cubic order, with RBCEV membrane proteins localized within phase-segregated nanodomains. Single-particle Raman analysis and enzymatic assays confirm molecular-level hybridization and preserved EV biofunctionality. Hybrid LCNPs also exhibit enhanced uptake in HEK293t cells. Mechanistic SAXS studies uncover that ethanol transiently stabilizes a swollen sponge-like intermediate, which mediates controlled fusion and acts as a structural template upon solvent removal, imparting long-lasting structural stability. This study elucidates the physicochemical mechanism of ethanol-guided hybridization between biogenic systems and soft nanostructured colloids, establishing design principles for structurally controlled nanohybrids with broad applicability in nanomedicine.

## Graphical abstract

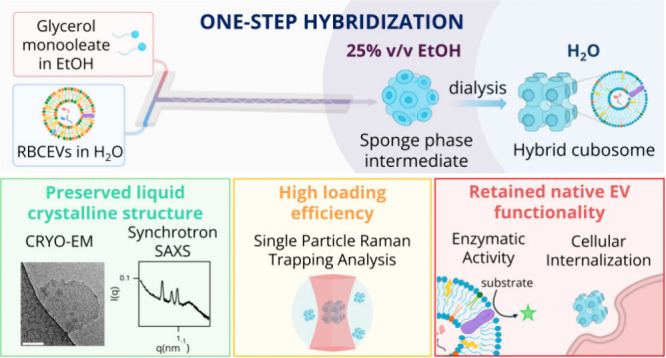

## 1 Introduction

Lipid-based nanoparticles (LNPs), ranging from liposomes to nonlamellar assemblies, have gained significant attention in recent years, as versatile carriers for delivering drugs, vaccines, and nutrients. Capable of protecting and transporting nucleic acids (NAs) to cells, LNPs unlocked unprecedented possibilities in gene delivery,^1^ including the emergency use authorization for anti-SARS-CoV-2 Pfizer and Moderna’s mRNA vaccines and the FDA approval of Onpattro, i.e., the first siRNA drug.^2,3^

Despite this undeniable success, LNP-based technology still faces persistent bottlenecks that substantially hinder a broader application in nanomedicine. Following endocytosis, LNPs typically struggle with poor endosomal escape, resulting in inefficient NA cytosolic release (∼2–3% of the cargo).^4^ Additionally, LNPs typically undergo quick opsonization in biological fluids, leading to fast clearance by phagocytes.^5^ Recognized as foreign bodies, they can also activate unpredictable innate immune responses, posing significant safety concerns. Finally, LNPs lack targeting abilities for specific organs or cell types and spontaneously accumulate in the liver,^9^ restricting their application to hepatic diseases and vaccines.^6^

Extracellular vesicles (EVs), membrane-bound vesicular structures released by cells and involved in intercellular communication,^7^ offer a promising avenue to overcome some of the limitations of LNP technology. EVs are nonimmunogenic and naturally present in body fluids as regulators of key physiological processes. Additionally, being “made by cells for cells”, they encode evolutionarily selected tissue-targeting strategies.^7,8^ However, when harnessed as delivery systems, EVs face major challenges, including limited scalability for large-scale production and inefficient drug loading.^9^

Hybridizing EVs with synthetic LNPs offers the opportunity of generating biogenic hybrids, which integrate the high-throughput manufacturing and high loading capacity of synthetic LNPs with the ability to cross biological barriers, low immunogenicity, and tissue-tropism of EVs.

Initial efforts in this direction explored hybridization strategies aided by physical stimulation, such a freeze–thaw cycles, sonication, or extrusion, to promote efficient fusion between EVs and LNPs.^9^ Despite promising results in terms of hybridization efficiency, these approaches are frequently associated with uncontrolled alterations in EV bioactivity, membrane integrity, and physicochemical properties,^9,10^ as well as potential losses of EVs intraluminal components^10–12^ and liposome-encapsulated cargos.^12–14^ Similarly, charge-based hybridization processes (mediated by permanently charged cationic lipids in LNPs) proved to negatively impact the native properties of EVs, leading to concentration-dependent toxicity and increased uptake by the mononuclear phagocyte system.^15,16^

Recent pioneering studies have demonstrated that efficient hybridization can also be achieved through passive coincubation of EVs with LNPs, reducing the impact on the native bioactivity and structural integrity of EVs.^17,18^ These approaches harness the intrinsic fusogenicity of a class of LNPs (nanoparticles with a nonlamellar liquid-crystalline internal structure (LCNPs)) to drive spontaneous membrane fusion with EVs, under simple mixing and physiological conditions.^17,18^ LCNPs also offer additional advantages, including enhanced cargo-loading capacity and mechanical robustness.^19^ Importantly, their highly curved bilayer organization promotes fusogenic interactions^20^ with biological membranes, a property that could be transferred to hybrid LNP/EV systems to facilitate cellular uptake and endosomal escape, thereby improving intracellular therapeutic delivery.

A major limitation of these approaches, however, is the lack of precise control over the fusion process, which remains highly sensitive to incubation parameters such as time, temperature, and pH,^24^ leading to structurally heterogeneous hybrids and particle aggregation under certain conditions. Critically, the original LCNP internal architecture (and its associated functional advantages, including high loading efficiency and enhanced endosomal escape) is partially or entirely lost upon hybridization with EVs.^17^

In this work, we introduce a single-step microfluidic strategy to engineer hybrid nonlamellar LNPs, specifically cubosomes, with prototypical EVs, namely, red-blood-cell-derived EVs (RBCEVs). Uniquely, in this approach, hybridization occurs *in situ* during LCNP formation via ethanol-assisted microfluidic preparation. Inspired by the microfluidic-based ethanol injection method used for mRNA encapsulation in LNP formulations,^3^ our process involves mixing an ethanol phase (containing synthetic lipids in monomeric form) with an aqueous phase containing EVs (in place of mRNA), followed by ethanol removal.

This process not only yields colloidally stable hybrid LCNPs with precise structural and compositional control but for the first time preserves the internal liquid-crystalline architecture of synthetic LCNPs following RBCEV hybridization. Synchrotron small-angle X-ray scattering (SAXS) and cryoelectron microscopy (cryo-EM) revealed hybrid nanoparticles featuring well-defined cubic internal structure alongside phase-separated amorphous nanodomains enriched in native RBCEV-associated proteins. Hybridization efficiency was quantified using single particle automated Raman trapping analysis (SPARTA), while enzymatic assays confirmed retention of the bioactivity of the RBCEV acetylcholinesterase membrane protein. Additionally, the hybrids exhibited enhanced cellular uptake in HEK293t cells.

From a mechanistic perspective, we demonstrate that the presence of ethanol during microfluidic is critical for both retaining the LCNP structure and enabling efficient encapsulation of RBCEV components.

This finding aligns with recent studies that increasingly highlight the role of ethanol in LNP formulation, though its mechanistic contribution remains largely unexplored. Ethanol is universally employed in LNP microfluidic preparation, where the water/ethanol volume ratio during mixing modulates LNP self-assembly and nucleic acid encapsulation.^3^ At the same time, the presence of ethanol in LNP preparations has been shown to destabilize lipid assemblies by altering membrane permeability and fluidity,^21,22^ promoting particle aggregation and potentially enhancing membrane fusion propensity.^23,24^ Beyond these empirical observations, however, the influence of ethanol on LNP-related processes, particularly particle fusion, remains poorly understood.

Addressing this gap, we here elucidate for the first time the mechanism of ethanol-assisted hybridization, revealing ethanol-mediated structural rearrangements within LNPs that drive RBCEV loading and impart long-term structural stability.

This proof-of-concept study establishes a high-throughput, scalable platform for generating hybrid biogenic LCNPs with high structural and compositional precision, potentially translatable to a broad range of synthetic/biogenic hybrids.

## 2 Materials and Methods

### 2.1 Materials

Glyceryl monooleate (GMO) was sourced from Croda (Cithrol GMO HP, minimum glyceryl monooleate content of 92%). DOPC (1,2-dioleoyl-*sn*-glycero-3-phosphocholine, ≥99%), sphingomyelin (≥99%), cholesterol (≥99%), DOPE-PEG(2000) carboxylic acid (≥99%), 18:1 Liss Rhod PE (≥99%) were obtained from Avanti Polar Lipids, Inc. (Alabaster, AL, USA). β-Bodipy FL C12-HPC (≥99%) was purchased from Invitrogen (Thermo Fisher Scientific). Ethanol absolute (≥99.9%) was purchased from Carlo Erba (Milan, Italy). Tetrachloroauric­(III) acid (≥99.9%), 5(6)-carboxyfluorescein (≥90%), Trizma base (tris­(hydroxymethyl)­aminomethane, ≥99.9%), calcium chloride (CaCl_2_, ≥97%), calcium ionophore (≥98%), 30% hydrogen peroxide (H_2_O_2_) stock solution, Tween-20, sodium dodecyl sulfate (SDS, ≥99%), glycerol (≥99%), 2-mercaptoethanol (≥99%), and sucrose (≥99.5%) were purchased from Sigma-Aldrich (St. Louis, MO, USA). Disposable and cell culture plasticware were purchased from Sarstedt Ag & Co. KG, (Nümbrecht, Germany). Sterile phosphate buffered saline (PBS) was purchased from Lonza (Basel, Switzerland). 0.45 μm Primo PES syringe filters used to filter PBS were purchased from Euroclone S.p.A. (Milan, Italy). All primary antibodies were purchased from Santa Cruz Biotechnology (Dallas, TX, U.S.) except for mouse-anti hemoglobin B, which was purchased from Abnova. HRP-conjugated secondary antibodies were purchased from Zymed (San Francisco, CA, US). Luminata Classic HRP Western substrate and PVDF membrane were purchased from Merck (Darmstadt, Germany). Acetylthiocholine chloride (≥99%) and 5,5′-dithio-bis­(2-nitrobenzoic acid) (≥98%) for enzymatic activity were purchased from Sigma-Aldrich (St. Louis, MO, USA). All products for cell culture were purchased from Thermo Fisher Scientific (Waltham, MA, USA) as well as the products used in confocal microscopy experiments and the LIVE/DEAD kit to study cell viability. Cells were obtained from the American Type Culture Collection (Manassas, VA, USA).

RBCEVs were produced and purified as described in section S1.1 of Supporting Information. Disposable, screw-cap, and bottle-cap assembly tubes for ultracentrifugation in the production and purification of RBCEVs were purchased from Beckman Coulter Inc. (Brea, CA, USA). 3D printing polypropylene filament (Ultrafuse PP) for chip manufacturing was purchased from BASF (Germany). If not stated elsewhere, Milli-Q-grade water was used in all preparations.

### 2.2 Microfluidic Preparation of AVs, LCNPs and LCNP/AVs or LCNP/RBCEVs Hybrids

Artificial vesicles (AVs) mimicking EVs, LCNPs, and hybrid LCNPs were prepared using a 3D-printed polypropylene microfluidic chip with two inlet channels, allowing simultaneous injection of an organic and an aqueous phase. The two inlets converge in a T-junction into the main channel, which presents a zigzag bas-relief structure along its length to induce chaotic mixing. The chip’s channels have a square cross-section of 1 mm, while the zigzag structure has a height of 500 μm. The total length of the main channel is 60 mm.^25^ A syringe pump system (Nemesys, CETONI GmbH, Germany) was used to inject the organic and aqueous phase. To prepare neat LCNPs, the organic phase consisted of a 30 mg/mL GMO solution in ethanol, while the aqueous phase only contained Milli-Q water. The water-to-ethanol flow rate ratio (FRR) during microfluidic preparation was set to 3:1 with a total flow rate (TFR) of 20 mL/min, resulting in an ethanol concentration of 25% (v/v) at the outlet. The flow conditions were found to minimize particle size and polydispersity, albeit varying the FRR from 3 to 4 and the TFR between 9 and 20 mL/min had only a minimal impact on the size and structure of the resulting LCNPs. Ethanol was subsequently removed by dialyzing the sample against Milli-Q water for 12 h, using a cellulose membrane with a molecular weight cutoff of 14 kDa (Sigma-Aldrich). Typically, around 4 mL of the sample was dialyzed against approximately 250 mL of Milli-Q water, which was replaced at least once during the process.

To prepare AVs, a lipid mixture composed of DOPC, sphingomyelin, and cholesterol (molar ratio 0.87:0.37:1 and total concentration, 16 mg/mL) was dissolved in ethanol. The organic phase was injected in one inlet, while Milli-Q water was simultaneously injected in the other inlet. We employed a FRR of 3:1 (water to ethanol) with a TFR of 20 mL/min, leading to an ethanol concentration in the final mix of 25% (v/v). The residual ethanol was removed as previously described, to obtain AVs at a 4 mg/mL lipid concentration. These flow conditions were selected as they yielded vesicles with an average diameter of 121 nm, within the typical size range of extracellular vesicles. Slower flow rates resulted in the formation of larger and more polydisperse vesicles.

To produce LCNP/AVs hybrids, the organic phase contained 30 mg/mL GMO in ethanol, and the aqueous phase consisted of a water dispersion of freshly prepared AVs, obtained as described above. The concentration of AVs in the aqueous phase varied from 0.3 mg/mL to 1.6 mg/mL, corresponding to 3% to 16% (w/w) with respect to GMO. The FRR and TFR were set at the same values described previously, leading to a residual ethanol concentration of 25% (v/v) in the main channel. LCNPs/RBCEV hybrid cubosomes were prepared following the same procedure, replacing AVs with RBCEVs in the aqueous phase, followed by ethanol removal via dialysis. The RBCEV concentration was expressed as weight percent relative to GMO and was calculated by weighing lyophilized aliquots of the RBCEV dispersion.

For SAXS measurements in Figure 2, samples were concentrated to 20 mg/mL using Amicon Ultra 0.5 mL centrifugal filters for 30 min at 3500 rpm, repeating the process multiple times if necessary.

For cellular uptake and viability assays, cubosomes were postfunctionalized with 0.5 mol % DOPE-PEG(2000) carboxylic acid to improve their colloidal stability in the cell culture medium. PEGylation was achieved by incubating the cubosome dispersion in water with a thin lipid film containing DOPE-PEG(2000) at 37 °C for 1 h, allowing spontaneous insertion of the PEGylated lipid into the cubosome membrane. Fluorescent labeling was performed during microfluidic preparation by codissolving the fluorescent lipid probe (0.1 mol % relative to GMO) with GMO in ethanol. β-BODIPY FL C12-HPC was used for flow cytometry and 18:1 Liss Rhod PE for confocal microscopy.

LCNPs used for single particle automated Raman trapping analysis (SPARTA;^26^ see section 2.7) were stabilized by incorporating 10 wt % Pluronic F127 relative to the total lipid content. The stabilizer was injected in the aqueous phase during the microfluidic preparation.

For cellular uptake, viability, SPARTA, and enzymatic assays, hybrid samples were prepared by incorporating either RBCEVs or AVs at a final concentration of 6.5 wt % relative to GMO.

### 2.3 Membrane Leakage Assay

Carboxyfluorescein-loaded vesicles (AVs@CF) were prepared employing a standard method of dry film rehydration. Briefly, DOPC, sphingomyelin, and cholesterol were dissolved in chloroform at molar ratios of 0.87:0.37:1, and the solvent was evaporated under a stream of nitrogen followed by overnight vacuum drying to obtain a lipid film. The film was then swollen and suspended in 10 mM Tris buffer (pH 7.4) containing 50 mM 5(6)-carboxyfluorescein (CF) by vigorous vortex mixing, achieving a final lipid concentration of 8 mg/mL. The resulting multilamellar liposome suspension was subjected to 10 freeze–thaw cycles and subsequently extruded 10 times through two stacked polycarbonate membranes with 100 nm pore size at room temperature using an extruder (Lipex Biomembranes, Vancouver, Canada). To remove unencapsulated carboxyfluorescein, 1 mL of the liposome suspension was passed through a disposable column prepacked with Sephadex G-25 (NAP-10 Columns, GE Healthcare UK Limited) in order to separate free dye from the CF-loaded liposomes. The liposomes were used immediately after purification.

Fluorescence intensities were then measured using a Fluoromax Plus (Horiba, Jobin Yvon) fluorescence spectrophotometer (excitation/emission: 492/514 nm). CF-loaded AVs at a concentration of 0.5 mg/mL were incubated with water/ethanol solutions at different ethanol concentrations for 1 h at room temperature and then diluted 1:200 in Tris buffer (pH 7.4) to minimize ethanol effects on fluorescence emission. To establish the baseline fluorescence, a control sample containing CF-loaded AVs in buffer alone was measured. Residual fluorescence was observed, likely due to incomplete removal of free CF. When CF-loaded AVs were treated with 0.5% Triton X-100 (TX) to disrupt the lipid bilayer and release all encapsulated CF, a 2-fold increase in fluorescence emission was observed. The percentage of CF leakage (Φ) for each ethanol condition was calculated using the following equation:

$$\mathrm{\% leakage}=\frac{I_{\mathrm{sample}}-I_{\mathrm{AVs}}}{I_{\mathrm{TX}}-I_{\mathrm{AVs}}}\times 100$$

where *I*_sample_ is the fluorescence intensity of AVs after 1 h incubation with water/ethanol and dilution 1:200 in tris, *I*_AVs_ is the fluorescence intensity of intact AVs (dispersed in buffer alone and diluted under the same conditions) at the same concentration, and *I*_TX_ is the fluorescence intensity of AVs after Triton X-100 treatment. The fluorescence intensities for each condition were measured in triplicate and averaged before analysis. The results were expressed as mean leakage percentages with standard errors.

### 2.4 Dynamic Light Scattering (DLS) and ζ-Potential

The hydrodynamic diameter, polydispersity index (PDI), and ζ-potential of AVs, RBCEVs, LCNPs, and hybrids were determined using dynamic light scattering (DLS) on a Malvern Zetasizer PRO Red Label (Malvern Panalytical, UK). Samples were diluted with Milli-Q water to 0.5 mg/mL lipid concentration and injected into DTS 1070 folded capillary cells (Malvern Panalytical, UK). The samples in the cells were stabilized at 25 °C, and the DLS measurement was recorded in triplicate at 173° (backscattering angle). ζ-Potential measurements were also recorded in triplicate.

### 2.5 Small-Angle X-ray Scattering

For SAXS measurements, 100 μL of sample was put into 1.5 mm thick borosilicate glass capillary tubes (WJM, Glas Müller GmbH, Berlin, German). Data were collected at the SAXS beamline of synchrotron radiation Elettra, Trieste (Italy) operated at 2.0 GeV with a beam energy of 8 keV (λ = 0.154 nm). SAXS curves were recorded using a Pilatus 3 1M detector in the *q*-range from 0.054 to 3.232 nm^–1^.

The cubic lattice parameter *d*_*c*_ was calculated from the maximum of each reflection (*q*_*hkl*_(*c*)) as

$$d_{c}=\frac{2\pi }{q_{hkl}\left(c\right)}\sqrt{\left(h^{2}+k^{2}+l^{2}\right)}$$

where *h*, *k*, and *l* are the Miller indices, as described previously.^27–30^ Water channel radius for cubic phases (*r*_*w*_(*c*))) was calculated using a geometrical model based on the curvature properties of the minimal surface, according to

$$r_{w\left(c\right)}=\left(\sqrt{-\frac{A_{0}}{2\pi \chi }}\cdot d_{c}\right)-l$$

with *A*_0_ and χ the surface area per volume and Euler–Poincaré characteristic of the cubic phase, and *l* the hydrophobic chain length (assumed to be 18 Å).^43^ For sponge (L_3_) phases (formed at 25 v/v% ethanol), the *d*-spacing *d*_s_ was estimated from the position of the lowest-*q* peak q_1_ as $d_{s}=2\pi /q_{1}\sqrt{2}$ (considering the Miller indices of the cubic *Im*3*m* or *Pn*3*m* phase obtained at slightly lower ethanol concentrations), and the water channel radius *r*_w(s)_ was calculated relative to the corresponding cubic *Im*3*m* or *Pn*3*m* phase (formed at 20 or 15 v/v% ethanol) using

$$r_{w\left(s\right)}=\frac{d_{s}}{d_{c}}\cdot r_{w\left(c\right)}$$

where *q*_1_(*s*) and *q*_1_(*c*) are the position of the lowest peak for the sponge phase and for the cubic phase, respectively.

### 2.6 Cryoelectron Microscopy

Cryo-EM data reported in Figures 1A and 3B were collected at imaging facility Unitech NOLIMITS (Università degli Studi di Milano, Milan, Italy). Prior to imaging, samples were vitrified at the Florence Center for Electron Nanoscopy (FloCEN), University of Florence. 3 μL of each sample was applied on glow-discharged Quantifoil Cu 300 R2/2 grids and plunge frozen in liquid ethane using an FEI Vitrobot Mark IV (Thermo Fisher Scientific). Blotting was performed for 1 s (blot force 1) under 100% humidity and 10 °C. Vitrified grids were then transferred to a Talos Arctica (Thermo Fisher Scientific) operated at 200 kV and equipped with a Ceta 16M detector (Thermo Fisher Scientific). Images in Figure 1A were acquired at a nominal magnification of 36k×, corresponding to a pixel size of 0.291 nm/pixel with a defocus of 3 μm. In Figure 3B, the first two images (from the left) were acquired at 28k× magnification (0.367 nm/pixel), while the last two images were acquired at 57k× magnification (0.182 nm/pixel); all were collected with a defocus of 3 μm.

**Figure 1:**
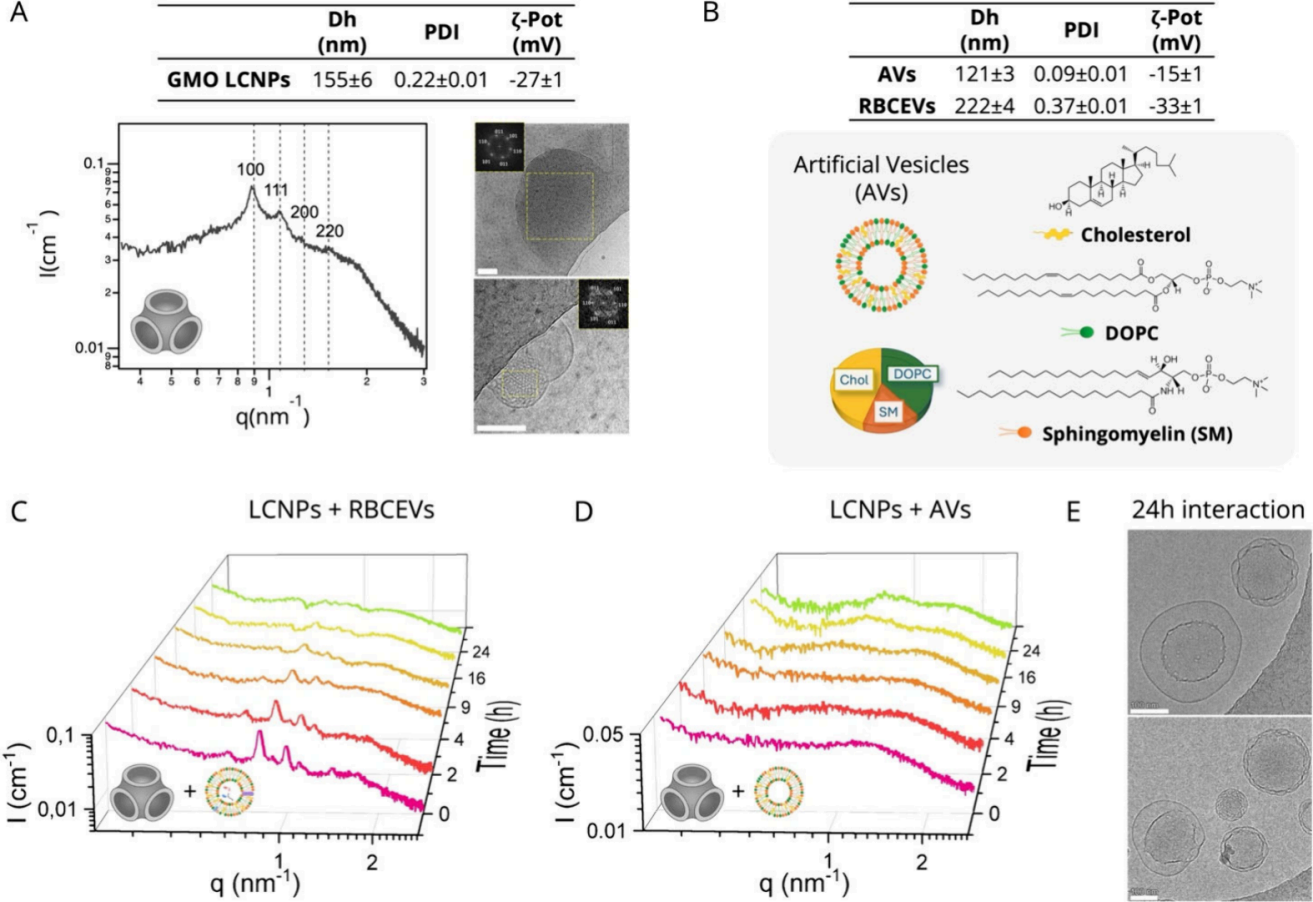
Characterization of LCNPs, AVs, and RBCEVs and their interaction. (A) Top: hydrodynamic diameter (Dh), polydispersity (PDI), and ζ-potential of LCNPs. Left: SAXS profile of LCNPs, where Bragg peaks are indexed with the corresponding Miller indices of the *Pn*3*m* phase and denoted by dashed lines. The internal structure of LCNPs is sketched on the bottom. Right: Representative cryo-EM images of LCNPs. The inset shows the fast Fourier transform of the yellow box area, identifying a liquid crystalline internal phase. Scale bar, 100 nm. (B) Top: Dh, PDI, and ζ-potential of AVs and RBCEVs. The bottom scheme illustrates the molecular composition of AVs. (C, D) SAXS profiles of LCNPs incubated with RBCEVs (C) and AVs (D) at different times at a vesicle content of 6.5 wt % relative to GMO LCNPs. All samples contained 10 mg/mL GMO. (E) Cryo-EM images of LCNP/AVs, after 24 h. Scale bar, 100 nm. All measurements were performed in Milli-Q water at 25 °C.

Cryo-EM data shown in Figures 1E and 3A were collected at the Cryo-Electron Microscopy Laboratory of ISASI-CNR in Naples. For sample preparation, 5 μL of each sample was applied to glow-discharged Quantifoil R 1.2/1.3 300 mesh Cu grids (Quantifoil, Großlöbichau, Germany) and flash frozen in liquid ethane using a Vitrobot mark IV (Thermo Fisher Scientific, Waltham, Massachusetts, USA) set at 100% humidity and 4 °C. Grids were blotted for 2 s following a 10 s wait time to promote nanoparticle distribution within the holes. Micrographs were acquired on a Glacios microscope (Thermo Fisher Scientific) operated at an accelerating voltage of 200 kV with a 50 μm C2 aperture. Images were acquired at nominal magnifications of 79k× (pixel size, 1.5 Å) and of 130k× (pixel size, 0.96 Å) in nanoprobe EFTEM mode with a total dose of 20 e/Å^2^ and a defocus value between −1.5 μm and −2 μm. A Falcon 4i direct electron detector was positioned after a Selectris energy filter (Thermo Fisher Scientific), operated in a zero-energy-loss mode with a slit width of 10 eV. The beam was set up in parallel illumination by diffraction on carbon foil to reduce artifacts. Astigmatism and coma were corrected using Sherpa software, and the energy filter was aligned to zero loss point before each grid area acquisition. Image collection and processing were performed using Smart EPU and Velox software (Thermo Fisher Scientific), respectively. Cryo-EM image analysis was performed through the ImageJ software.

### 2.7 SPARTA Single Particle Composition Measurements by Raman Spectroscopy

SPARTA measurements were performed on the SPARTA AGIS I benchtop instrument (SPARTA Biodiscovery, UK). Instrument control and data acquisition were performed in SPARTA Control v1.1.3. A 100 μL aliquot of particle suspension (1 × 10^10^ to 1 × 10^12^ particles mL^–1^) was dispensed onto a SPARTA sample slide and positioned under the objective lens. All measurements were carried out at ambient temperature (20 ± 1 °C). Individual particles were held for 10 s per trap while their Raman signal was measured, after which the laser shutter was closed for 1 s to release the trapped particle and allow diffusion of a new particle into the confocal volume. Blank Dulbecco’s phosphate-buffered saline (DPBS, Thermo Fisher Scientific) was measured for 20 acquisitions at 10 s and the average was used for background subtraction.

The data were plotted and analyzed using in-house SPARTA Discovery software v.1.1.3. A spectral response correction was applied, cosmic spikes were removed using limit 40, empty traps were removed through thresholding and a background subtraction of 95% was applied. All spectra were then truncated to an ROI of 416–1800 cm^–1^, followed by a baseline correction with smoothness factor 7 and differential order 2. The data was then smoothed using a filter with order 2 and window 7 before being normalized by setting the area under each spectrum to 1.

After preprocessing, a subpopulation was identified in GMO-containing samples which was distinguished by differences in the spectral region 850–1050 cm^–1^ and made up 7–15% of the total particles analyzed. As this subpopulation interfered with analysis of the protein peak, it was removed by thresholding the preprocessed data on the 965 cm^–1^ peak to leave the main particle population, which was used then for analysis in this work.

### 2.8 Acetylcholinesterase Activity Assay

Acetylcholinesterase activity was assessed by standard procedures.^31^ Briefly, 20 μL of native RBCEVs, RBCEVs passed in the microfluidic device (RBC-EV microfluidic), bare LCNPs, and LCNP/RBCEVs were diluted in 60 mL of PBS without calcium and magnesium and incubated with 1.25 mM of acetylthiocholine and 0.1 mM of 5,5′-dithio-bis­(2-nitrobenzoic acid) in a final volume of 1 mL. The incubation was carried out in 96-well plates at 37 °C, and the change in absorbance at 415 nm was followed at 0, 10, 20, 30, 60, and 150 min. Absorbance was measured with the spectrophotometer model 680 Bio-Rad (USA). Quantitative analysis of the mean acetylcholinesterase activity was performed by calculating the ratio of the absorbance at 415 nm of native RBCEVs to that of LCNP/RBCEV hybrids, averaged across all time points. Three independent measurements were conducted for each time point.

### 2.9 Western Blot for RBCEVs and LCNP/RBCEVs Hybrid Cubosomes Markers

RBCEV, RBC-EV microfluidic, LCNPs and LCNP/RBCEVs aliquots were boiled for 5 min at 95 °C in reducing SDS sample buffer (80 mM Tris, pH 6.8, 2% SDS, 7.5% glycerol, and 0.01% bromophenol blue) supplemented with 2% 2-mercaptoethanol. Samples were normalized based on their RBCEV and GMO content before being loaded onto a 10% acrylamide/bis­(acrylamide) gel for protein separation via SDS–PAGE (130 V, fixed voltage for 1 h and 20 min at RT). Proteins were then transferred onto a PVDF membrane (100 V, fixed voltage, 1 h at 4 °C) and blocked overnight in PBS + 0.05% Tween-20 (PBST) + 5% fat-free milk. The PVDF membranes were then incubated for 90 min at RT under agitation with the following primary antibodies, diluted 1:1000 in PBST with 1% fat-free milk: mouse anti-BAND3, mouse anti-hemoglobin B and mouse anti-acetylcholinesterase. After primary antibody incubation, the membranes were washed three times for 10 min each with PBST under agitation, followed by a 1 h incubation with HRP-conjugated rabbit anti-mouse secondary antibodies at a 1:3000 dilution. The membranes were then washed three additional times for 10 min each with PBST under agitation. Detection was performed using 2 mL/membrane of Luminata Classic HRP Western substrate, which was incubated with the membranes for 5 min in the dark. Chemiluminescence was captured using a G:Box Chemi XT imaging system, acquiring three cumulative exposures of 3 min each, with the camera shutter fully open.

### 2.10 Cell Culture

Hek 293t (human embryonic kidney) cells (American Type Culture Collection, Manassas, VA, USA) were cultured in Dulbecco’s modified Eagle’s medium (DMEM) (Thermo Fisher Scientific, Waltham, MA, USA) supplemented with 1% penicillin/streptomycin solution and 10% fetal bovine serum (FBS) (ThermoFisher Scientific, Waltham, MA, USA). Cell cultures were incubated at 37 °C, 5% CO_2_ in a humidified atmosphere. The cultures were grown until 90% confluency.

#### 2.10.1 Confocal Microscopy Uptake Studies

Hek 293t cells were plated on 18 mm glass coverslips in 12-well plates at 100 000 cells per well. 24 h after plating, cells were incubated for 24 h with unlabeled LCNP, LCNP labeled with Liss Rhod PE (LCNP-Rhod), LCNP-Rhod/RBCEV and LCNP-Rhod/AV in DMEM. Subsequently, they were incubated with 5 μg/mL WGA-488 (ThermoFisher Scientific) for 30 min to specifically label the plasma membrane. Ten minutes before the end of this incubation time, cells were incubated with Hoechst 33342 (ThermoFisher Scientific) dye at a concentration of 10 μg/mL in order to stain the nuclei of the cells. After the incubation, cells were washed with PBS, mounted on a custom chamber for imaging and the medium was replaced with Leibovitz’s L-15 (ThermoFisher Scientific) before imaging, a medium designed for supporting cell growth in the absence of CO_2_ equilibration.

The analysis was performed with a Nikon Eclipse TE300C2 LCSM (Nikon) equipped with a Nikon Plan Apo λ 100× 1.49 NA oil immersion objective and with Coherent CUBE (diode 405 nm), Melles Griot (Argon 488 nm) and Coherent Sapphire (Sapphire 561 nm) lasers. Emission filters for imaging were 452/45 nm, 514/30 nm, and 595/60 nm.

The analysis of LCNP-Rhod, LCNP-Rhod/RBCEV, LCNP-Rhod/AV was performed after excitation at 561, 488, and 405 nm. For each sample, optical sections at median planes of the cells were taken (1024 × 1024 pixels) using subsaturation settings and all of them were kept constant for each analysis (laser power, detector gain, and pinhole diameter). Images were processed and analyzed with the Fiji software.

#### 2.10.2 Flow Cytometry Uptake Studies

Hek 293t cells were plated in 12-well plates at 100 000 cells per well. 24 h after plating, cells were incubated with LCNPs, LCNP/RBCEVs, LCNP/AVs for 2, 4 and 24 h. After incubation cells were washed with PBS and harvested in DMEM after mild trypsin treatment. Flow cytometry was performed by using a Accuri-C6 flow cytometer (BD Biosciences, Lake Franklin, NJ, USA) equipped with 488 and 640 nm lasers. β-BODIPY FL C12-HPC was excited using the 488 nm laser, and the emitted fluorescence was collected through a 530/15 nm band-pass filter.

Data were analyzed using the BD Accuri-C6 software and Origin (Pro) version 2022b (OriginLab Corporation). The cellular debris was excluded from the quantification by gating cells on the forward scatter area/side scatter area (FSC/SSC). Approximately 15 000 events were acquired for each sample.

### 2.11 Cell Viability

Hek 293t cells were plated in 12-well plates at 100 000 cells per well. 24 h after plating, cells were incubated with LCNPs, LCNP/RBCEVs, and LCNP/AVs for 24 h. Subsequently, cells were incubated for 30 min at RT with the LIVE/DEAD kit (ThermoFisher Scientific) according to the manufacturer’s recommendations. With this kit, viability is measured by staining with green-fluorescent calcein-AM to indicate intracellular esterase activity. Green-fluorescent calcein-AM was excited using the 488 nm laser, and the emitted fluorescence was collected through a 530/15 nm band-pass filter. Then, cells were washed with PBS and harvested in DMEM after mild trypsin treatment. Flow cytometry was performed by using a Accuri-C6 flow cytometer (BD Biosciences, Lake Franklin, NJ, USA). Data were analyzed using the BD Accuri-C6 software.

## 3 Results and Discussion

### 3.1 Production of LCNP/RBCEVs Hybrids by Passive Incubation

As a reference standard, LCNPs/RBCEV hybrids were formed employing the state-of-the-art preparation method, i.e., simple incubation of LCNPs and RBCEV aqueous dispersions at room temperature. This strategy leverages the intrinsic fusogenicity of LCNPs to induce spontaneous fusion with RBCEVs under physiological conditions.^17,18^ The so-formed hybrids were then compared to those produced with the new one-step microfluidic hybridization approach described in section 3.2.

Synthetic LCNPs were produced using glycerol monooleate (GMO), an FDA-approved lipid classified as “generally recognized as safe” (GRAS) for use in food and pharmaceuticals. GMO is known to spontaneously form nonlamellar liquid crystalline structures through spontaneous self-assembly in aqueous media.^29^ LCNPs were prepared using a microfluidic-based ethanol injection method,^5^ (see section 2.2), widely employed in LNPs formulation, which yields an aqueous dispersion of LCNPs after ethanol removal following dialysis.

DLS and ζ-potential analyses (Figure 1A, top table) show that LCNPs have an average hydrodynamic diameter of 160 nm and a negative surface charge in Milli-Q water.

The SAXS profile of LCNPs (Figure 1A, left) displays typical Bragg reflexes of particles with a liquid-crystalline internal phase having a cubic *Pn*3*m* crystallographic space group, namely, cubosomes. This structure is bicontinuous, featuring a single lipid bilayer of negative interfacial curvature, which divides the 3D space into two sets of interwoven aqueous nanochannels. From SAXS analysis, we estimated a lattice parameter (*d*) of such arrangement of 10.1 nm, as described in section 2.5. Cryo-EM analysis (Figure 1A, right) shows LCNPs with homogeneous, highly ordered internal liquid-crystalline structure and a globular morphology. Additionally, LCNPs feature a bilayer-like arrangement at the lipid–water interface, consistent with previous observations of cubosomes assembled without polymeric steric stabilizers.^32^

On the other side RBCEVs (see section S1.1 of Supporting Information production and preparation) are characterized by an average hydrodynamic diameter of ∼222 nm, high size polydispersity and negative surface charge in Milli-Q water (Figure 1B, top table). In this study, RBCEVs were systematically compared to artificial RBCEV mimics (referred to as artificial vesicles (AVs)) which are composed exclusively of lipids and lack both transmembrane and luminal proteins. This simplified design allowed us to isolate and investigate separately the specific contributions of membrane lipids and proteins to the fusion and hybridization processes with LCNPs. Moreover, free from the polydispersity, morphological complexity, and compositional heterogeneity characteristic of biological EVs, AVs with their precisely controlled size and composition served as reference standards, providing a robust platform for mechanistic studies. The lipid composition of artificial vesicles (AVs) (DOPC, sphingomyelin, and cholesterol (0.87:0.37:1 molar ratio) (see bottom scheme in Figure 1B)) was selected to retain the hallmark feature of EV membranes from mammalian sources, i.e., the enrichment in sphingomyelin and cholesterol, as compared to parental cells.^33^ AVs were prepared following standard microfluidic preparation procedures for liposomes (see section 2.4), yielding monodisperse vesicles with ∼120 nm hydrodynamic size and slightly negative surface ζ-potential (Figure 1B, top table).

To investigate their spontaneous interaction, we mixed LCNPs with either RBCEVs or AVs at a 6.5% weight ratio (wt %) relative to GMO LCNPs, in Milli-Q-water and room temperature. We recorded SAXS profiles of LCNP/RBCEVs (Figure 1C) and LCNP/AVs (Figure 1D) at different incubation times (from 5 min to 30 h). Under these conditions, the scattering profile is dominated by LCNPs, with a negligible scattering contribution from vesicles (see Figure S1).

After 5 min of incubation with RBCEVs, the scattering pattern of the mixed system corresponds to the original *Pn*3*m* cubic phase, with a significant lattice swelling (from 10.1 to 11.0 nm). An additional peak appears at lower scattering vector (q) values, possibly indicating the coexistence of the *Pn*3*m* phase with a more swollen liquid-crystalline structure. As incubation time increases, the scattering features of the cubic phase gradually decrease in intensity and completely disappear after ∼16 h, indicating a loss of the internal liquid-crystalline structure. The interaction and fusion with LCNPs occur significantly faster for AVs, leading to complete disruption of the cubic architecture within 5 min. At longer incubation times (∼16 h), structural rearrangements occur, highlighted by the emergence of a broad peak at intermediate q values, likely indicating a disordered and heterogeneous organization.^34^ We imaged these structures by cryo-EM after 24 h of incubation (Figure 1E), revealing core–shell particles with pronounced heterogeneity in both core and shell size and structure. Additionally, residual fragments of liquid-crystalline structures bridging hemifused vesicles were occasionally observed (Figure S2).

The faster interaction observed with AVs compared to RBCEVs suggests that vesicle-derived lipids (rather than proteins) play a key role in promoting fusion and hybridization with LCNPs. This interpretation is further supported by the higher lipid content the LCNP/AVs hybrid: in LCNP/AVs, AVs contribute 6.5 wt % entirely as lipids, whereas in LCNP/RBCEVs formulations, the same 6.5 wt % of RBCEVs includes both lipids and proteins. Therefore, the stronger and faster interaction with AVs is likely due to a larger content of vesicle-derived lipids, suggesting that proteins in RBCEVs contribute less significantly to the interaction.

Overall, these findings are consistent with recent studies demonstrating efficient LCNP-mediated hybridization.^17,18^ However, this efficiency comes at a cost: a complete, or near-complete, loss of the internal liquid-crystalline structure, along with its associated functional advantages, such as high loading capacity and enhanced endosomal escape. Moreover, the resulting particles exhibit substantial size and structural heterogeneity (see additional cryo-EM images in Figure S2), which severely limits the ability to control structure–function relationships. Remarkably, this variability likely reflects a similarly high heterogeneity in particle composition, resulting in uneven functionality and biological responses across the particle population, thereby possibly compromising the consistency and predictability of the biological performance.

### 3.2 Single-Step Microfluidic Engineering of LCNP/RBCEVs Hybrids

To address the limitations of passive coincubation approaches, we developed a novel microfluidic-based platform for hybridizing LCNPs with EVs. Microfluidic mixing offers unprecedented control and reproducibility of the hybridization conditions, while providing a scalable, manufacturing-compatible solution for large-scale production. Our strategy takes inspiration from the well-established microfluidic ethanol-injection method used to formulate mRNA-loaded LNPs, in which an ethanol phase containing synthetic lipids is rapidly mixed with an aqueous phase containing mRNA, followed by ethanol removal.^3^ Building on this principle, we introduce a fundamentally new application of the ethanol injection method: the “*in situ*” hybridization of LCNPs with EVs, in which LCNP formation and membrane fusion with EVs occur simultaneously.

Our method employs a 3D-printed polypropylene microfluidic chip with two inlet channels for the coinjection of an ethanol phase (containing synthetic LCNP lipids) and an aqueous phase (containing RBCEVs), as illustrated in Figure 2A. The two streams meet at a T-junction and enter a main channel with a zigzag bas-relief structure designed to induce chaotic mixing. Unlike traditional LNP-mRNA formulations, here RBCEVs replace mRNA as the aqueous-phase cargo. The two phases are mixed at a total flow rate of 20 mL/min and a 3:1 water-to-ethanol ratio, yielding a final ethanol content of 25% v/v in the central channel. Ethanol was subsequently removed by 24 h dialysis in ultrapure water to ensure an ethanol residual content <5% v/v, in line with pharmaceutical-grade LNP formulation standards.^3^

**Figure 2:**
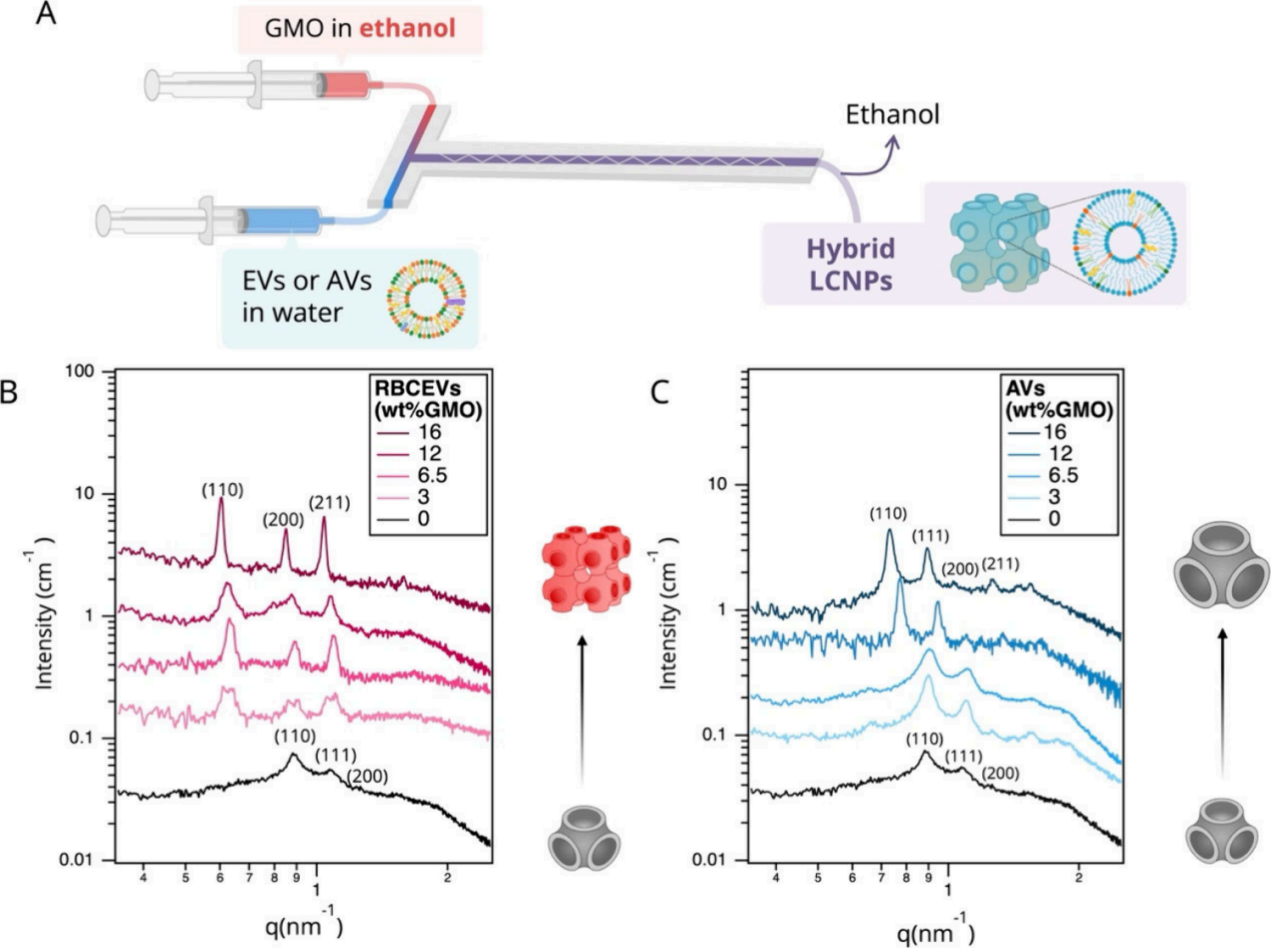
Production and characterization of LCNP/RBCEVs and LCNP/AVs. (A) Schematic illustration of the microfluidic platform employed to produce LCNPs/RBCEV and LCNP/AVs. (B, C) SAXS profiles of LCNP/RBCEVs (B) and LCNP/AVs (C) obtained at different RBCEVs or AVs wt % (relative to GMO) in Milli-Q water and 25 °C. The Bragg peaks are indexed with the corresponding Miller indices of either a *Pn*3*m* or a *Im*3*m* phase. All samples were measured after 4 days from preparation. The insets in (B) and (C) illustrate LCNP structural rearrangements upon RBCEV and AV loading. All measurements were performed at a GMO concentration of 20 mg/mL.

Critically, the solubility of GMO (present as a monomer in the ethanol phase) drops upon contact with the aqueous stream within the main channel, triggering self-assembly, which thereby occurs concurrently with RBCEV hybridization.

We tested LCNPs hybridization with both RBCEVs and AVs, at varying GMO/vesicles weight ratios, by modulating the vesicle concentration in the aqueous phase, while fixing the one of GMO in ethanol at 3.66 wt %. DLS analysis highlighted that LCNP/RBCEVs hybrids are colloidally stable, exhibiting a slightly larger hydrodynamic diameter than bare LCNPs and a similarly negative ζ-potential (Table 1). LCNP/AVs hybrids show a more pronounced increase in hydrodynamic diameter (∼200–230 nm) compared to original LCNPs, yet retaining moderately low polydispersity and negative ζ-potential. Importantly, both LCNP/RBCEVs and LCNP/AVs hybrids exhibited enhanced colloidal stability compared to neat LCNPs, which typically undergo rapid aggregation in the absence of stabilizers such as Pluronic F-127 or PEGylated lipids.

**Table 1:** ^a^

sample	phase	*d* (nm)	*R*_w_ (nm)	Dh (nm)	PDI	ζ-Pot (mV)
LCNPs	*Pn*3*m*	10.1	2.2	155 ± 6	0.22 ± 0.01	–27 ± 1
LCNP/RBCEVs (wt %)						
3.0	*Im*3*m*	13.9	2.4	229 ± 3	0.09 ± 0.02	–39 ± 1
6.5	*Im*3*m*	14.2	2.5	215 ± 8	0.05 ± 0.02	–27 ± 1
12	*Im*3*m*	14.3	2.6	224 ± 4	0.06 ± 0.02	–27 ± 1
16	*Im*3*m*	14.7	2.7	201 ± 2	0.17 ± 0.01	–35 ± 1
LCNP/Avs (wt %)						
3.0	*Pn*3*m*	10.0	2.2	180 ± 8	0.10 ± 0.01	–30 ± 1
6.5	*Pn*3*m*	9.9	2.2	160 ± 5	0.14 ± 0.03	–27 ± 1
12	*Pn*3*m*	11.5	2.7	164 ± 5	0.09 ± 0.01	–26 ± 1
16	*Pn*3*m*	12.2	3.0	153 ± 4	0.12 ± 0.01	–32 ± 1

a Phase structure, lattice parameter (*d*), radius of water channels (*R*_w_), hydrodynamic diameter (Dh), polydispersity (PDI), and *ζ*-potential for LCNPs, and hybrid LCNP/RBCEVs or LCNP/AVs, prepared at varying RBCEVs and AVs wt % (relative to GMO). For SAXS measurements, the concentration of GMO was 20 mg/mL. For DLS and ζ-potential measurements, samples were diluted to 0.5 mg/mL GMO.

SAXS analysis revealed that the hybridization with RBCEVs leads to a significant structural rearrangement even at the lowest RBCEV content (3 wt %) (Figure 2B). Specifically, hybridization leads to a phase transition in the LCNPs scaffold from the cubic *Pn*3*m* phase to the cubic Im3m phase, which, while still exhibiting a bicontinuous cubic architecture, is characterized by increased lattice swelling and higher water content (Table 1). Increasing the RBCEVs content results in a progressive swelling of the Im3m lattice parameter (from 13.9 to 14.7 nm) and the water channel radius (from 2.4 to 2.7 nm). Crucially, while RBCEVs induce significant structural rearrangements (reflecting efficient interaction and loading), the cubic liquid-crystalline internal structure remains fully preserved even after 4 days (black solid profile in Figure S3) and up to 1 week (red dashed profile in Figure S3), with no detectable changes in scattering intensity. Hybridization with AVs induces similar effects, although to a lesser extent (Figure 2C). For these systems, the original *Pn*3*m* structure is retained across all GMO/AVs weight ratios, albeit its lattice parameter progressively increases from 10.1 to 12.2 nm as AV content rises. This is consistent with the integration of AV components (cholesterol, DOPC, and sphingomyelin) into the GMO matrix, which promote a shift toward less negative membrane curvature values.^35,36^

Compared to LCNP/AVs hybrids, RBCEVs incorporation leads to more pronounced lattice swelling at equivalent weight ratios, resulting in a *Pn*3*m*-to-Im3m phase transition that AV lipids alone are insufficient to trigger. This is likely due to additional protein components unique to EVs, which are not present in their synthetic mimics. RBCEVs typically contain over 300 different proteins,^37^ including both soluble proteins enclosed within the lumen (e.g., hemoglobin) and membrane-associated proteins that may be adsorbed onto, associated with, or embedded within the lipid bilayer.^38^ These surface proteins play a key role in determining the natural targeting and circulation behavior of RBCEVs.^39^ Given their considerable size relative to lipids, the inclusion of both luminal and membrane-associated proteins within the LCNPs can significantly affect lipid packing and alter the final particle structure.^40^

We further characterized the morphology of hybrids though cryo-EM, with representative micrographs of LCNP/AVs and LCNP/RBCEVs shown in Figure 3A and Figure 3B, respectively, alongside fast Fourier transforms (FFT) as insets highlighting the liquid-crystalline internal structure.

**Figure 3:**
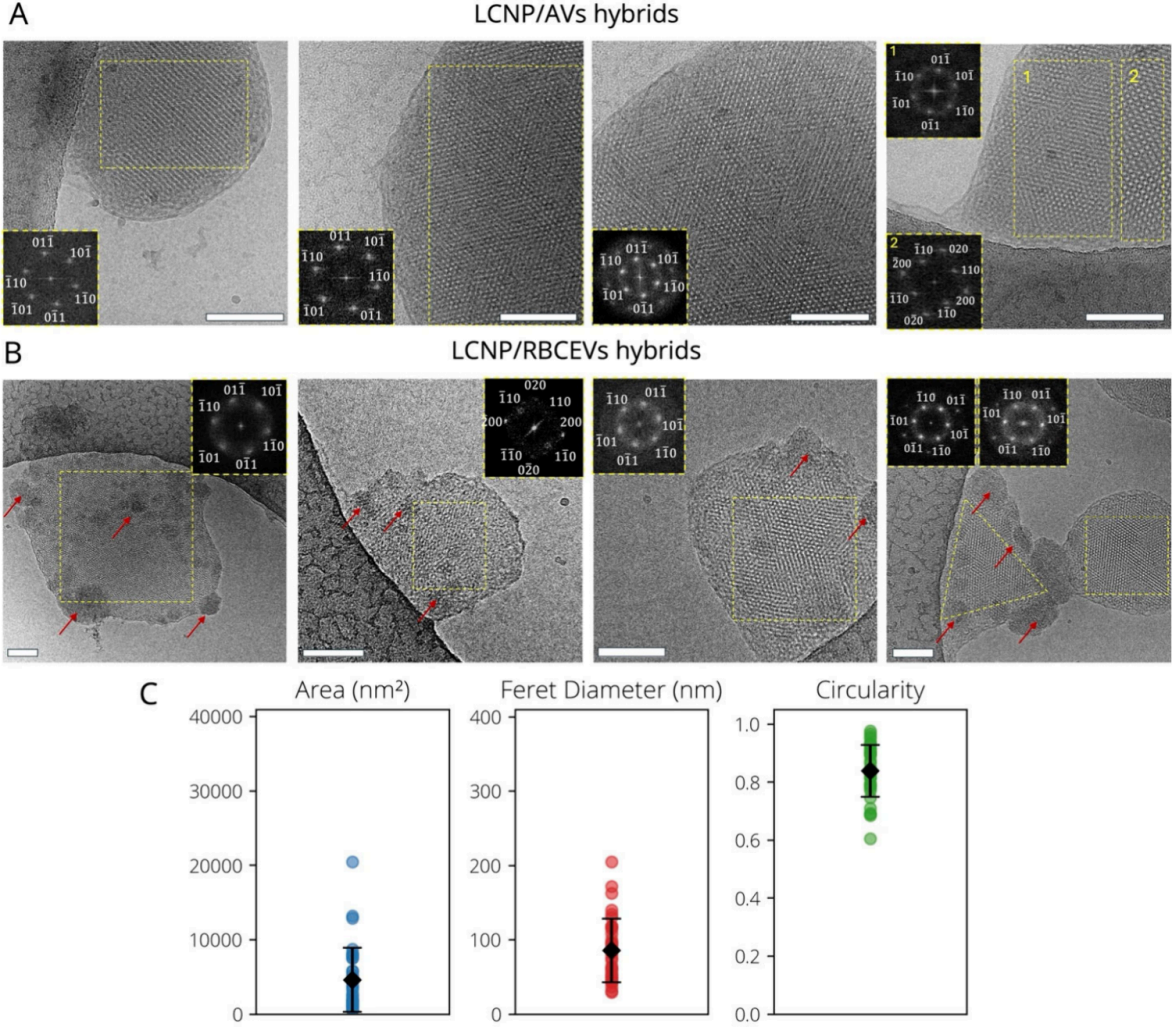
Morphological characterization of LCNP/AVs and LCNP/RBCEVs. (A, B) Representative cryo-EM images of LCNP/AVs (A) and LCNP/RBCEVs (B), with insets showing the fast Fourier transform of the yellow box area, identifying a liquid crystalline internal phase. Samples were imaged 7 to 10 days from preparation. Scale bar: 100 nm. (C) Scatterplots obtained from cryo-EM image analysis through the ImageJ software, showing the average area, diameter, and circularity of phase-separated electron-dense domains in LCNP/RBCEVs. These data were obtained analyzing a total of 67 particles with diameters ranging from 29 to 205 nm.

LCNP/AVs display a rather spherical morphology and sizes larger than those measured by DLS (Table 1), likely due to some aggregation occurred during the 10-day storage prior imaging and grid blotting. In agreement with SAXS analysis, hybrids feature a liquid crystalline internal structure, whose lattice parameter was calculated through FFT (Figure S4). In a minority of particles (upper right image, Figure 3A), analysis revealed liquid-crystalline phases with distinct lattice parameters at the single-particle level, suggesting coexistence of the *Pn*3*m* phase with other liquid crystalline structures (feature not observed at the particle ensemble-level trough SAXS).

In contrast, LCNP/RBCEVs exhibit irregular morphologies, with liquid-crystalline domains coexisting alongside amorphous, electron-dense regions, resulting in particles with multiple internal phase separations. These features are absent in LCNP/AVs, suggesting that they arise from the hybridization with biological components present in RBCEVs but absent in synthetic vesicles. Notably, the size of these electron-dense domains is significantly smaller than that of whole RBCEVs (Dh ∼ 222 nm) (Figure 3C), indicating that they likely result from spontaneous phase segregation of native EV proteins rather than full vesicle encapsulation. Supporting this hypothesis, RBCEV lumen is known to contain hemoglobin, which (due to its Fe content) might appear more electron-dense than lipid regions. Interestingly, these domains exhibit a characteristic near-spherical morphology, with an average circularity of 0.85 (Figures 3C and S5), reminiscent of controlled protein segregation occurring in protein-enriched “rafts” in biological membranes.

These findings demonstrate that the ethanol-assisted microfluidic platform developed here enables the production of colloidally stable LCNP/EV hybrids with precise control over composition and internal structure. The method allows for easy tuning of EV content, which in turn finely modulates the structural features of the resulting hybrids. Unlike conventional hybridization approaches, this strategy preserves the native liquid-crystalline architecture of LCNPs, crucial for maintaining their structure-dependent functional properties.

To fully exploit this approach, we then investigated the hybridization efficiency and the functional properties of the resulting hybrids to assess whether the native features of RBCEVs are successfully transferred to LCNP/RBCEVs hybrids.

### 3.3 Hybridization Efficiency and Functional Properties of LCNP/RBCEVs Hybrids

To evaluate the hybridization efficiency, we employed an emerging experimental method, single particle automated Raman trapping analysis (SPARTA).^26^ Using a combination of laser optical trapping and Raman spectroscopy, this technology enables detailed compositional analysis of single nanoparticles in a fully automated, nondestructive label-free process. This technique has recently been applied to study the composition of EVs, LCNPs, and AVs.^41,42^ By simultaneously detecting signals from synthetic lipids and biological components within hybrids (having distinctive Raman fingerprints), this method yields the distribution of hybridization across the LCNP population.

To this purpose, SPARTA measurements were performed on RBCEV, LCNP, and LCNP/RBCEVs samples. In a typical measurement, ∼200 particles were individually optically trapped while their Raman spectra were recorded. Examining the mean spectra ± standard deviation, calculated across all individual particles measured within a sample, shows strong, distinct signals from the pure LCNP and RBCEV samples (Figure 4A). The mean spectrum of neat LCNP displays Raman signals characteristic of the GMO lipid, including a strong peak at 1650 cm^–1^ deriving from the stretching vibration of unsaturated bonds (C=C, C=O) present within the molecule, as well as peaks at 1300 cm^–1^, from the bending vibrations of CH_2_ bonds in the lipid tail, and at 1445 cm^–1^, deriving from bending of CH_2_/CH_3_ bonds. In comparison, RBCEVs display Raman peaks associated with cholesterol (located at 700 cm^–1^ and associated with the C–C backbone vibrational modes of the sterol ring), choline lipid headgroup (715 cm^–1^, representing C–N stretching vibrations), and protein components (1000 and 1660 cm^–1^) across the sample. In particular, the protein-related band at 1660 cm^–1^ corresponds to the peptide bond vibration of the amide I band (α-helix configuration), with a possible contribution from C=C stretching from unsaturated fatty acid chains in phospholipids within RBCEVs. In contrast, the signal at 1000 cm^–1^ originates from the ring-breathing mode of the aromatic ring in phenylalanine residues and is insensitive to protein conformation.

**Figure 4:**
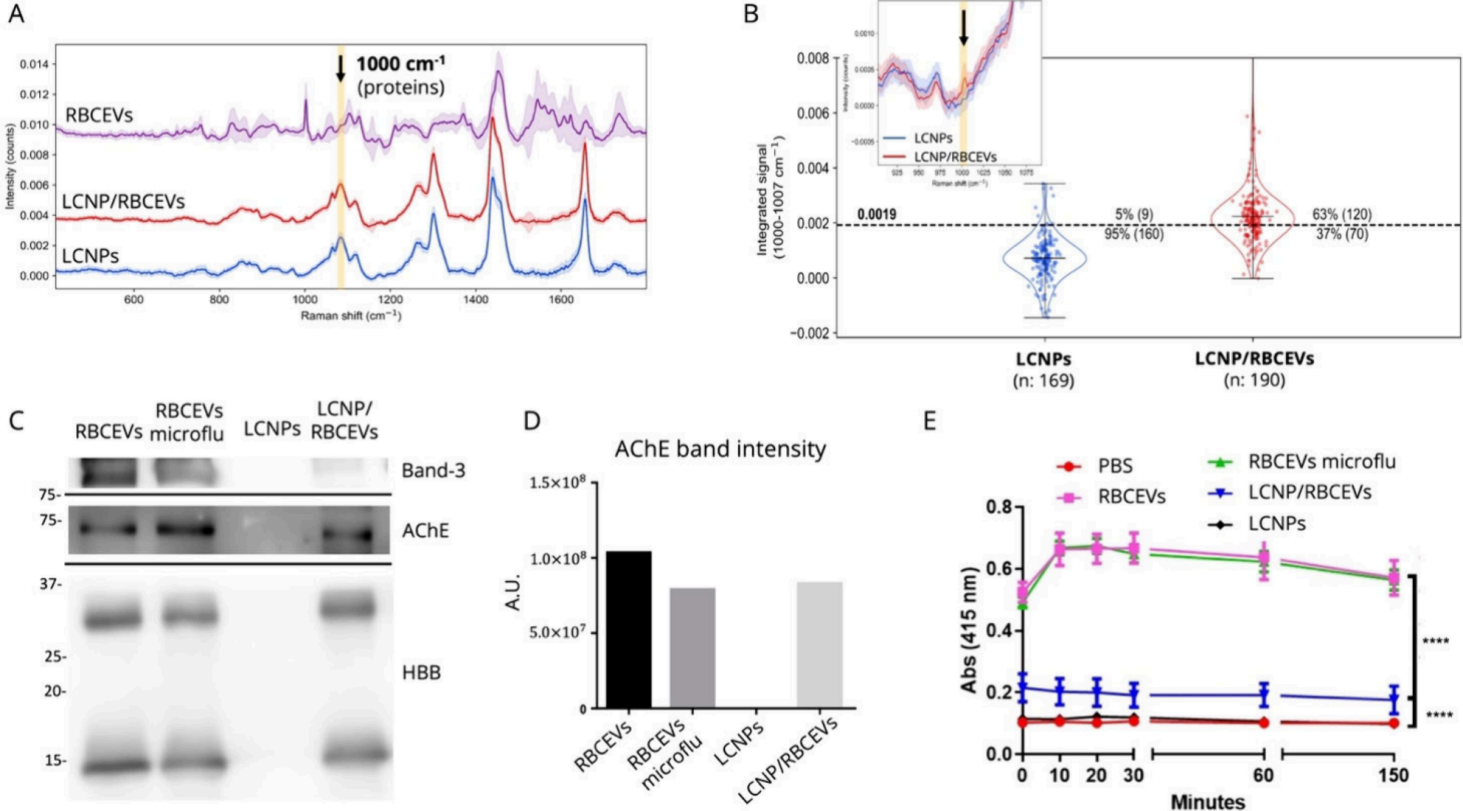
Hybridization efficiency, biochemical characterization, and enzymatic activity of LCNP/RBCEVs. (A) Mean Raman spectra ± standard deviation, calculated across all individual particles measured within samples of native RBCEVs, bare LCNPs, and LCNP/RBCEVs. (B) Integrated Raman signal intensity in the 1000–1007 cm^–1^ range for each particle contained in pure LCNP and LCNP/RBCEVs preparations. (C) Western blot analysis of RBCEV, RBCEV processed with the microfluidic device (RBCEV microflu), LCNP alone (LCNPs), LCNP and RBCEV after microfluidic processing (LCNP/RBCEVs hybrids) for the presence of Band-3, acetylcholinesterase (AchE), and hemoglobin B (HBB). Equal amounts of all samples (15 μL) were loaded on a 12.5% acrylamide/bis­(acrylamide) gel. (D) Representative signal band quantification of the acetylcholinesterase (AchE) protein in all samples described above. A.U.: arbitrary unit. (E) Enzymatic activity of achetylcolinesterase (AchE) in all the samples described above after 10, 20, 30, 60, and 150 min of incubation with acetylthiocholine at 37 °C. ****Student *t* test, *p* value of <0.0001 PBS vs LCNP/RBCEVs and LCNP/RBCEVs vs RBCEVs.

Crucially, the protein-related Raman signal is also detected in the mean spectra of LCNP/RBCEVs hybrids, while it is not present in bare LCNPs, indicating successful hybridization (Figure 4A). Therefore, the area of the peak at 1000 cm^–1^, from phenylalanine residues in proteins (Figure 4A, highlighted region), was used to assess incorporation of RBCEVs into LNPs.

In this analysis, a particle was classified as hybridized if its protein signal (at 1000 cm^–1^) exceeded that of 95% of the pure LCNP population (Figure 4B). This thresholding analysis revealed that 63% of the particles in the LCNP/RBCEVs sample exhibited protein/lipid hybridization signatures, while the remaining 37% displayed only GMO-derived signals. This assessment was based on a total of 190 analyzed particles. Notably, no pure RBCEVs were detected within the LCNP/RBCEVs population (that is, no particles showed exclusively RBC-related signals without the GMO Raman fingerprint), indicating that the entire RBCEV population, present in substoichiometric amounts relative to LCNPs, was fully consumed through hybridization. This agrees with the cryo-EM data, showing no evidence of residual RBCEV presence.

To assess which protein components were successfully transferred to the hybrids, we performed Western blot analysis on LCNP/RBCEVs samples, using native RBCEVs and bare LCNPs as controls. In addition, RBCEVs exposed to microfluidic mixing with ethanol under the same conditions used for hybrid preparation (RBCEV-microflu) were also analyzed to rule out potential protein loss caused by ethanol-induced membrane restructuring and permeabilization.

Specifically, we assessed the presence of proteins commonly found in RBCEVs,^37^ i.e., the membrane protein Band-3,^43^ the membrane-bound enzyme acetylcholinesterase (AChE),^44^ and hemoglobin B. All three proteins were detected in samples containing RBCEVs, while no signal related to these proteins was observed in the pure GMO control (Figure 4C). Notably, the band intensities were consistent across all RBCEV-containing formulations, and quantification of the AChE band (Figure 4D) confirmed comparable enzyme levels in all cases. These results indicate that the microfluidic preparation does not lead to significant protein loss, and that protein content is largely preserved from native RBCEVs to the hybrids.

To investigate how the hybridization affects the enzymatic activity, we measured AChE activity using a colorimetric assay based on the hydrolysis of acetylthiocholine.

Native RBCEVs and RBCEV-microflu samples (Figure 4E, pink and green lines) exhibited comparable activity, indicating that ethanol exposure and microfluidic processing do not impair enzyme bioactivity. In contrast, a significant reduction in AChE activity was observed in LCNPs/RBCEV hybrids (Figure 4E, blue line) compared to native RBCEVs, while the enzyme remains functionally active, with activity levels significantly above those of the negative controls (Figure 4E, red and black lines). Quantitatively, the mean AChE activity in the hybrids is reduced to 32 ± 4% of that measured in native RBCEVs.

This reduction occurs despite comparable AChE protein levels across all RBCEV-containing samples, as confirmed by Western blot analysis, and cannot therefore be attributed to protein loss during hybrid fabrication. Additionally, because the RBCEV-microflu sample (exposed to the same ethanol and microfluidic conditions) retains full enzymatic activity, the observed decrease cannot be attributed to compromised bioactivity arising from protein denaturation during the ethanol-based fabrication process. Instead, the decreased apparent activity is more plausibly explained by partial confinement of AChE within the internal bicontinuous structure of the cubic lipid nanoparticles. LCNPs with cubic symmetry exhibit an exceptionally high bilayer/water interfacial area (∼400 m^2^/g),^45^ which provides extensive surface availability for enzymatic reactions—significantly higher than that of vesicular systems (∼5–10 m^2^/g, based on typical phospholipid headgroup areas and molecular weights). However, unlike vesicles, where the lipid membrane interfaces directly with bulk water, most of this interfacial area in LCNPs is internal, embedded within a network of confined aqueous nanochannels. This nanoconfined environment may influence the conformation and catalytic efficiency of AChE, a dimeric enzyme tethered to the RBC membrane via a glycosylphosphatidyl­inositol (GPI) anchor, with an approximate size of approximately 4.5 nm × 6.0 nm × 6.5 nm. In the hybrid LCNP/RBCEVs, which exhibits an *Im*3*m* symmetry with water channels around 5 nm in diameter (Table 1), the enzyme likely experiences steric confinement that limits substrate access to the active site located ∼2 nm deep within a narrow gorge of the protein. Yet the persistence of measurable enzymatic activity well above negative controls indicates that AChE remains functionally active within the hybrid nanostructure. Comparable activity losses have been observed in lipid cubic mesophases where enzyme size approaches or exceeds the pore size.^46,47^ These studies demonstrate that enzymatic activity can be efficiently restored by minimally increasing the water channel size of cubic assemblies, reaching levels comparable to those of the unconfined enzyme once the channels exceed the enzyme’s diameter. Such swelling can be readily achieved through the inclusion of molecular additives (e.g., sugar esters) that expand the cubic structure.^46^

### 3.4 Cellular Internalization

To assess the effect of LCNP hybridization with RBCEVs, we performed cell internalization studies, via confocal microscopy analysis using rhodamine-labeled systems (see section 2.2), including bare LCNPs labeled with rhodamine (LCNPs-Rhod), or hybrid systems labeled with the same dye (i.e., LCNPs-Rhod/AVs, and LCNPs-Rhod/RBCEVs). HEK293t cells were incubated with 0.1 mg/mL of each formulation for 24 h. Then, the plasma membrane was stained with the dye WGA-488, a marker that binds specifically to the sialic acid present in the cell membrane. After washing and replacing the culture medium, images were acquired (Figure 5A). Confocal imaging revealed significant internalization of all LCNP types into the cytoplasm of HEK293t cells after 24 h (Figure 5B).

**Figure 5:**
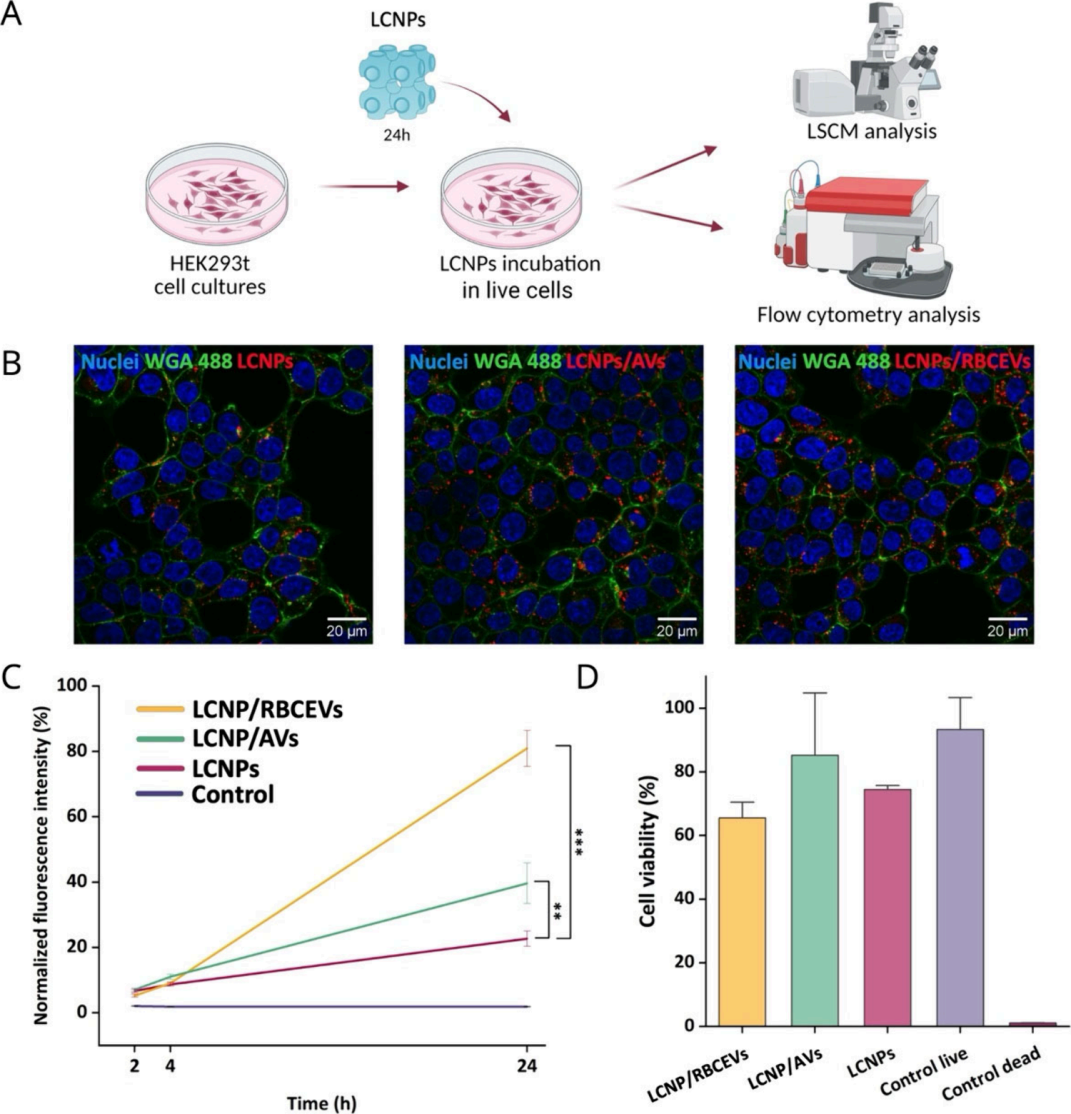
Cellular uptake and cell viability studies. (A) Live cells experiment workflow (created with Biorender.com). (B) Representative confocal images of HEK293t cells incubated for 24 h with LCNPs-Rhod, LCNPs-Rhod/AVs, or LCNPs-Rhod/RBCEVs at a final concentration of 0.1 mg/mL and then with 5 μg/mL WGA-488 for 30 min to specifically label the plasma membrane. Red, Rhod-LCNPs; green, WGA-488; blue, Hoechst for labeling of cell nuclei. Scale bar: 20 μm. (C) Quantification of cellular internalization by measuring the mean fluorescence intensity from the LCNPs labeled with BODIPY by flow cytometry. Fluorescence intensity values were normalized to the maximum value observed at 24 h. Error bars: SE. Asterisks indicate significant differences using Kruskal–Wallis ANOVA test followed by Dunn’s multiple comparison post hoc test (**p < 0.05, ***p < 0.001). (D) Cell viability measured by flow cytometry with the LIVE/DEAD assay as mean fluorescence intensity. Cells were incubated with 0.1 mg/mL of each of the 3 formulations.

To quantitatively compare the uptake of the different formulations, flow cytometry analysis was employed. For this purpose, LCNPs were fluorescently labeled with the BODIPY fluorescent dye (see section 2.2). Cells were incubated with the labeled LCNPs, LCNP/RBCEVs and LCNP/AVs (0.1 mg/mL) for 2, 4, and 24 h. Results showed a progressive increase in fluorescence intensity over time, with a significantly higher uptake observed for the LCNP/RBCEVs compared to LCNPs alone, particularly at 24 h (Figure 5C).

Cell viability was assessed by flow cytometry using a LIVE/DEAD assay. Cells were incubated with LCNPs, LCNP/RBCEVs, and LCNP/AVs at a final concentration of 0.1 mg/mL for 24 h. Fluorescence was detected in the FL1 channel (derived from calcein-AM, a component in the LIVE/DEAD assay that labels live cells), and viability was quantified based on fluorescence intensity corresponding to viable cells (Figure 5D). Most cells across all samples displayed strong fluorescence, indicating a high overall viability rate, with only a moderate reduction in the samples incubated with LCNP/RBCEVs.

Overall, these results indicate that functionalization with AV, and even more so with RBCEVs, enhances the cellular uptake of LCNPs, as demonstrated by both confocal microscopy and flow cytometry (Figure 5B,C). Moreover, AV and RBCEV functionalization does not significantly affect cell viability, with all formulations being well tolerated by the cells. This is further supported by confocal images (Figure 5B), which show preserved nuclear morphology, typical cell shape, and clear evidence of cell division.

Cells exhibiting high mean fluorescence intensity (FL1 channel showing the fluorescence derived from the green-fluorescent calcein-AM) correspond to fully viable cells (control live), while lower fluorescence intensity indicates less viable cells (control dead); fluorescence intensity values were normalized to the maximum value; error bars are SE.

### 3.5 Mechanism of Hybridization: The Role of Ethanol

Overall, these findings demonstrate that this microfluidic approach enables the production of colloidally stable LCNP/EV hybrids with high hybridization efficiency, while preserving the native bioactivity of EVs (section 3.3). Importantly, hybridization with RBCEVs enhances cellular uptake compared to unmodified LCNPs (section 3.4). Compared to the state of the art, this approach offers unique advantages: it allows precise control over hybrid composition and internal structure, enabling fine-tuning of the embedded EV content (section 3.2). Additionally, its single-step, high-throughput nature and reliance on microfluidics make the process readily scalable for large-scale manufacturing.

Crucially, unlike existing hybridization strategies, this ethanol-assisted method preserves the internal liquid-crystalline architecture of LCNPs (section 2.2), a key feature for retaining their structure-dependent functional properties, such as high cargo-loading capacity and fusogenicity toward endosomal membranes.

However, key questions remain unanswered. Why are these hybrids structurally stable while those obtained with passive co-incubation, which possess the same final composition, exhibit disruption of the liquid crystalline structure? The simplest explanation lies in the different preparation methods, which involves a component, EtOH, which is then removed from the sample, but could mediate the formation of these different structures? If so, what is the underlying mechanism by which ethanol influences the formation, morphology, and long-term stability of these hybrids? Gaining this fundamental knowledge is critical to rationally optimize formation of hybrids with desired properties and to expand the applicability of this method for producing structurally defined hybrids across a range of nanoparticle and vesicle types.

During microfluidic hybridization, vesicles in the aqueous phase are mixed with GMO dissolved in ethanol, resulting in a 25% v/v ethanol concentration within the main mixing channel. Ethanol is known to influence lipid solubility, self-assembly in water/ethanol mixtures, and membrane structure, altering lipid hydration and acyl chain order.^48–50^ Since both LCNPs and vesicles are lipid-based systems with membrane-like architectures, the presence of ethanol during mixing likely impacts both components. To elucidate these effects, we first investigated the influence of ethanol on the vesicle structure and LCNP self-assembly independently and then examined its role in modulating their mutual interaction.

Using artificial EV mimics, we evaluated the physicochemical properties (size (Figure 6A), membrane integrity (Figure 6B), and rigidity (Figure 6C,D)) of AVs upon exposure to increasing ethanol concentrations. To this end, we mixed AVs with a water/ethanol solution with varying ethanol % v/v, to reach a final ethanol content in the mix in the range 0–90% v/v.

**Figure 6:**
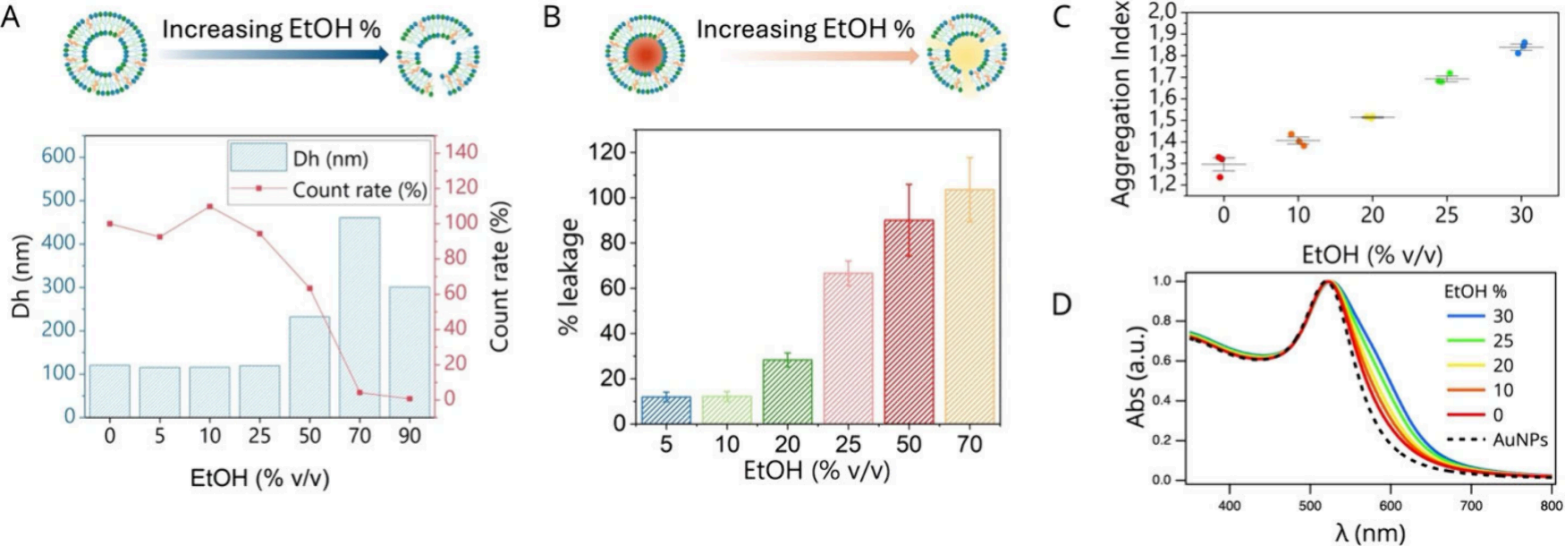
Ethanol effects on AV size, membrane permeability, and stiffness. (A) Double plot displaying AV hydrodynamic diameter (right) and average scattering intensity (left) as a function of ethanol concentration (% v/v) in the medium. (B) CF leakage (%) from AVs at increasing ethanol concentrations, calculated from fluorescence intensity and normalized to the signal at 0% ethanol (set as 0% leakage) and 0.5% Triton X-100 (set as 100% leakage). (C) Aggregation indexes for AuNPs incubated with AV prepared at varying ethanol concentrations, calculated from the UV–vis spectra in (D). All the measurements were performed in Milli-Q water, at 25 °C, at least in triplicate.

DLS results showed that vesicle size and average scattering intensity remain stable up to 25% ethanol (v/v), suggesting that moderate ethanol concentrations do not induce vesicle disruption (Figure 6A). This is further supported by the lack of significant changes in polydispersity within the same ethanol range (Figure S6). From 50% ethanol onward, vesicle destabilization and disruption occur, as marked by significant increases in size and polydispersity, along with a drastic reduction in the average scattering intensity (Figure 6A). Since the ethanol content during microfluidic mixing is 25% v/v (below the disruption threshold), we conclude that hybridization occurs in the presence of intact vesicles. To probe the effect of ethanol on vesicle membrane integrity, we used a leakage assay, based on the fluorescent dye carboxyfluorescein (CF).^51^ CF was encapsulated in the aqueous core of AVs at a self-quenching concentration, and unencapsulated dye was removed by purification (see section 2.3). Under these conditions, any leakage of CF resulting from membrane permeabilization can be detected by an increase in the fluorescence intensity. In the absence of ethanol, fluorescence remains low, indicating limited membrane permeability (Figure S7). AVs were then mixed with water/ethanol solutions at increasing ethanol concentrations, and fluorescence intensity was measured after 60 min of incubation (Figure S7).

In the 5–25% v/v ethanol range, a gradual increase in fluorescence was observed, reflecting progressive CF leakage (Figure 6B). This suggests that although AVs remain structurally intact, moderate ethanol concentrations (comparable to those used in microfluidic hybridization) still significantly enhance membrane permeability, promoting dye release. At ethanol concentrations above 25% v/v, nearly 100% of the dye is released, likely due to complete vesicle disruption.

To assess potential changes in AV membrane fluidity within the 0–25% v/v ethanol range (where vesicles remain intact), we employed a nanoplasmonic assay recently developed by our group,^52–54^ previously shown to effectively probe membrane stiffness in both synthetic liposomes and biological vesicles. This method leverages the spontaneous clustering of plasmonic gold nanoparticles (AuNPs) on vesicle membranes, a process finely modulated by membrane stiffness. AuNP clustering alters their plasmonic response, which is readily detectable via UV–vis spectroscopy and quantified using an aggregation index (A.I.) derived from the spectral profile.^54,55^ The A.I. correlates directly with membrane stiffness through an empirical sigmoidal relationship, enabling straightforward assessment of vesicle rigidity variations. Using this approach, we observed a progressive increase in AuNP clustering on vesicles prepared with increasing ethanol concentrations, reflected by a corresponding rise in the A.I. (Figure 6C, calculated from UV–vis spectra in Figure 6D). This trend qualitatively indicates a continuous decrease in AV membrane stiffness across the 0–25% v/v ethanol range, revealing that even moderate ethanol levels can induce significant membrane fluidification.

These findings suggest that the 25% v/v ethanol present during hybridization preserves vesicle integrity, preventing the substantial loss of EV luminal cargo typically observed with physical stimulation-based methods that induce membrane rupture, such as sonication, electroporation, or extrusion.^11–13^ While leaving vesicles intact, however, the presence of ethanol alters lipid packing (enhancing membrane permeability and fluidity) possibly reducing the energy barrier for fusion with LCNPs and promoting hybridization.

These results are in line with previous studies, where ethanol was found to increase the area per lipid molecule and decrease the bilayer thickness, thus making membranes more permeable to small molecules.^56,57^ This effect was also previously connected to increased membrane fusogenicity, promoting formation of stalk intermediates (the linking of two leaflets of adjacent membranes before the formation of a fusion pore), leading to membrane fusion.^58^ This process could happen through a transient breakage in the two lipid leaflets, which decreases the tensile strength of the lipid membrane and its rigidity.^59^

To complete this information, we further investigate ethanol effects on LCNPs self-assembly and hybridization with vesicles.

In our microfluidic approach, GMO is injected as a monomer fully dispersed in ethanol; upon mixing, the ethanol concentration is diluted to 25% v/v in the main channel (where hybridization occurs) triggering GMO self-assembly due to reduced lipid solubility. However, previous studies on LNP-mRNA suggest that the final structure of LNPs only forms after complete ethanol removal, which can drive additional structural rearrangements.

To understand the mechanism driving this process and its impact on hybridization with RBCEVs, we analyzed the structure of hybrids immediately after microfluidic formation (at 25% v/v ethanol) and at various time points during dialysis (0–24 h) against ultrapure water. Ethanol content at each stage was quantified using refractive index measurements (see section S1.3 and Table S2). For comparison, we also performed the same analysis on pure LCNPs formed in the absence of vesicles.

The SAXS profile of GMO in 25% v/v ethanol (in the absence of vesicles) does not display the Bragg peak pattern characteristic of the *Pn*3*m* phase (Figure 7A), observed after complete ethanol removal (Figure 1A). Instead, a broad peak located at *q* = 0.6 nm^–1^ emerges together with a very-low intensity bump at 0.98 nm^–1^. These features are compatible with a sponge (L_3_) phase arrangement,^60^ previously observed in bulk ternary systems composed of GMO, water, and water-miscible solvents such as dimethyl sulfoxide, propylene glycol, polyethylene glycol, and ethanol.^61,62^ This arrangement presents a lower degree of order than cubic phases (thereby is often described as a “melted” cubic phase) and is composed of randomly connected bilayer networks at extremely low concentrations in aqueous media.^63^

**Figure 7:**
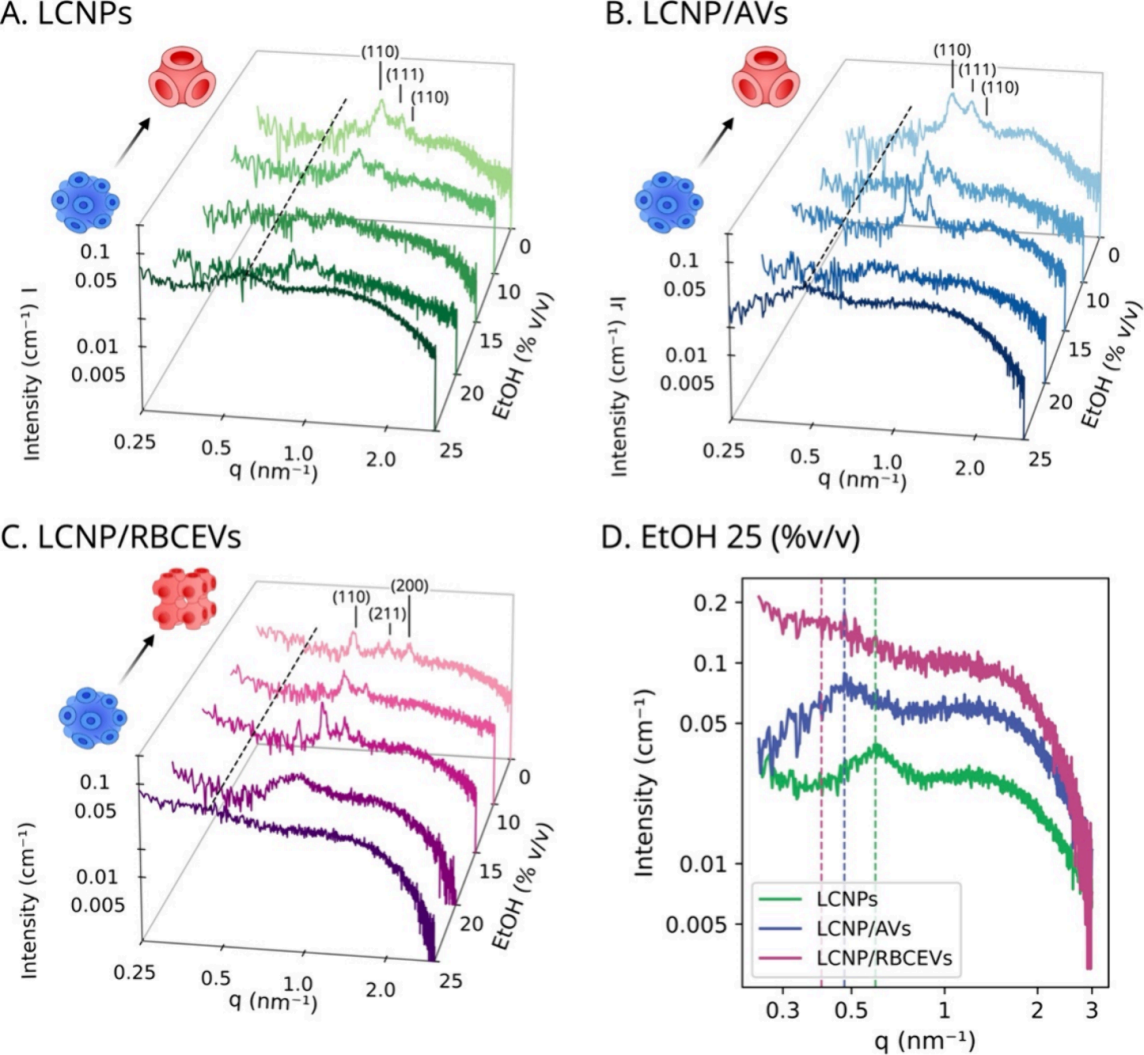
Ethanol effects on LCNP formation and hybridization. (A–C) SAXS profiles of pure LCNPs (A), LCNP/AVs (6.5 wt %) (B), and LCNP/RBCEVs (6.5 wt %) (C) in water/ethanol mixtures at varying ethanol % v/v, mimicking the structural evolution of LCNP upon dialysis to remove ethanol, following microfluidic preparation. The single peak of the sponge phases is marked through dashed lines, while peaks of the cubic *Pn*3*m* and *Im*3*m* are indexed with the corresponding Miller indices. (D) SAXS profiles of LCNPs, LCNP/AVs and LCNP/RBCEVs at 25% v/v ethanol concentration, showing a progressive swelling of the sponge phase from LCNP to LCNP/RBCEVs, marked by a shift of the sponge peak at lower *q*-values. The position of the sponge peak is marked by dashed vertical lines for each sample. All measurements were performed at a GMO concentration of 10 mg/mL.

In L_3_ phases, the peak at lower-*q* values corresponds to the L_3_ cell–cell correlation distance (connected to water channels diameter) and the higher *q*-peak to the lipid bilayer thickness. Due to the high bilayer dilution of L_3_, such peaks typically exhibit low scattering intensities, particularly when confined within nanoparticles.^64^

By considering the lower-*q* peak value, we calculated a lattice spacing and water channel radius of 14.8 and 2.2 nm, respectively. In line with literature on similar L_3_ systems,^60,64^ these values are significantly larger than those observed for GMO in the cubic phase (Table 1), indicating higher swelling of water channels. This is a key feature of sponge phases, where the large aqueous pores have been shown to uniquely favor the encapsulation and concentration of large biomolecules^65,66^ (outperforming other lipid structures), finding application in drug and food delivery, protein crystallization,^67^ and artificial organelles.^68^

The presence of this arrangement in our system likely arises from ethanol-induced membrane expansion and structural disordering (effects observed in both synthetic^69^ and natural membranes^59^), resulting in a melted state of the cubic phase. Reducing the ethanol content to 20% v/v triggers a phase transition from the L_3_ phase to the *Pn*3*m* cubic phase, likely due to decreased solubility of GMO acyl chains, resulting in a more compact and less hydrated lattice structure. Further ethanol reduction causes a gradual contraction of the *Pn*3*m* phase, which reaches its minimum equilibrium lattice parameter only at 0% v/v ethanol (Table S1).

A closely similar behavior was observed for LCNP/AVs hybrids (Figure 7B), where the L_3_ phase also forms in the presence of AVs. However, in this case, the L_3_-to-*Pn*3*m* phase transition occurs at lower ethanol concentrations, with *Pn*3*m* Bragg peaks becoming visible only from 15% v/v ethanol onward. In the case of LCNP/RBCEVs, even more pronounced differences were observed (Figure 7C). At 25% v/v ethanol, a broad, low-intensity shoulder appears instead of a distinct peak, indicating the presence of an extremely swollen network with high bilayer dilution. Reducing the ethanol content to 20% v/v induces the formation of an L_3_ phase, which further transitions into a cubic *Im*3*m* phase at 15% v/v. This cubic structure then progressively contracts as the ethanol content decreases from 15% to 0% v/v (Table S1).

These results indicate that both AVs and RBCEVs promote the formation of highly swollen sponge (L_3_) networks, stabilizing them and delaying their transition to cubic phases as ethanol is gradually removed. Further supporting this observation, the L_3_-associated peak shifts progressively to lower q values from pure LCNPs to LCNP/AVs and LCNP/RBCEVs (Figure 7D), indicating increasingly swollen networks. Quantitative analysis of the SAXS data reveals a systematic expansion of both lattice parameters and aqueous pore sizes, increasing from 14.9 and 2.4 nm for LCNPs to 18.7 and 3.1 nm for LCNPs/AVs, and further to 22.8 and 6.1 nm for LCNPs/RBCEVs.

In the case of LCNPs/AVs, the observed L_3_-phase swelling is primarily driven by the incorporation of lipids from AVs. Among them, phosphatidylcholines and sphingomyelin contained in AVs, have larger and more hydrophilic headgroups compared to GMO, favoring structures with reduced interfacial curvature and increased hydration. Although differences in lipid composition between AVs and native RBCEVs are expected due to the high compositional complexity of RBCEVs, AVs nonetheless provide a valuable protein-free reference system for isolating lipid-driven contributions to mesophase swelling.

For LCNPs hybridized with native RBCEVs, this lipid-induced swelling is likely further amplified by the presence of vesicle-associated proteins. Owing to their size and amphiphilic (for transmembrane proteins) or hydrophilic (for luminal proteins) character, these proteins can introduce additional steric constraints at the lipid–water interface, promoting stabilization of highly swollen L_3_ phases and favoring bicontinuous networks with larger aqueous pores compared to lipid-only systems. This interpretation is consistent with recent reports on sponge-phase lipid nanoparticles interacting with proteins such as myoglobin, where protein incorporation was shown to shift interfacial curvature from slightly negative toward near-zero values, facilitating sponge-to-lamellar transitions.^64^ In line with these studies, our results indicate that the presence of proteins in RBCEVs enhances mesophase swelling beyond that achieved by lipids alone, leading to larger pore sizes and increased interfacial curvature of the bicontinuous network.

Importantly, similar sponge-phase intermediates evolving into ordered hybrid LCNPs upon ethanol evaporation can also be generated *ex situ* by simply mixing GMO and RBCEVs at the same weight ratios in a glass vial, with both components prepared in 25% v/v ethanol (Figure S8A). Under these conditions, ethanol and water mix completely, replicating the mixing efficiency achieved in-chip through the zigzag geometry of the microfluidic channel, which induces chaotic advection.^39^ In contrast, when solvent mixing is incomplete (e.g., mixing GMO in ethanol with RBCEVs in water in a vial, without chaotic mixing), effective contact between synthetic lipids and RBCEVs is hindered, ultimately preventing hybrid LCNP formation (Figure S8B).

Overall, these results highlight that hybridization occurs prior to the formation of the final cubic LCNP structure, specifically when LCNPs are assembled in a highly swollen, large-pored sponge (L_3_) phase (Figure 8). This marks a key distinction from existing passive coincubation approaches, where fully formed LCNPs in water interact with EVs postassembly. Due to its open and flexible architecture, the L_3_ phase can readily accommodate structural perturbations from lipid and protein inclusions, allowing efficient EV encapsulation with minimal structural rearrangements. Moreover, ethanol-induced increases in EV membrane permeability and deformability likely lower even further the energetic barrier for fusion and encapsulation (Figure 8).

**Figure 8:**
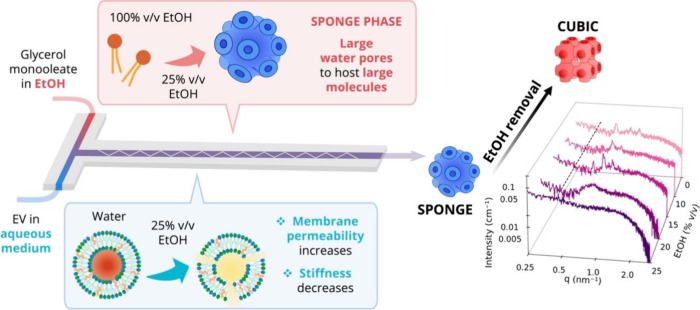
Schematic representation of ethanol-mediated hybridization between LCNPs and EVs. In the microfluidic system, GMO dissolved in ethanol and EVs in aqueous medium are rapidly mixed, yielding a final ethanol concentration of 25% v/v. Under these conditions, GMO assembles into a sponge (L3) phase with large, disordered water channels, while ethanol simultaneously increases EV membrane permeability and fluidity. Upon ethanol removal, the system transitions into a more ordered cubic phase.

In contrast, energetic costs required to incorporate EVs into the cubic phase of preformed LCNPs in 100% water (characterized by narrower lattice spacing, smaller water channels, and lower membrane fluidity) are likely higher, leading to the destabilization of this arrangement and the transition to core–shell vesicular structures.

Importantly, these core–shell structures with no internal liquid-crystalline phase are not formed when the sponge phase is lost via ethanol removal. Instead, the 3D network gradually contracts, culminating in the formation of a stable cubic phase at 0% v/v ethanol (Figure 8). This final state remains colloidally stable for at least a week and retains a signature of EV loading, evidenced by a larger lattice parameter compared to bare LCNPs. These final ethanol-free hybrids likely represent metastable structures, kinetically trapped and templated by the sponge phase ancestor. The hybridization conditions (mediated by ethanol) play a decisive role in defining the final structure, even after ethanol has been completely removed. In this sense, the transient ethanol-induced sponge phase acts as a structural “imprinting” stage that leaves a lasting impact on the final architecture of the hybrid particles.

Similar metastable systems, where a transient intermediate phase templates the final structure, are also observed in nature, where non equilibrium conditions and kinetic trapping provide access to functional structures which are normally precluded by thermodynamic equilibrium. As an example, ethanol has been found to induce nonbilayer structures within the membrane interior in biological membranes, which have a long-lived character. These nonlamellar arrangements are often accompanied by lipid mixing between opposing membrane leaflets and lipid reorganization, resulting in irreversible alterations to membrane structure and compositional asymmetry,^48,59^ changes that may have significant implications for membrane-associated biological functions.

## 4 Conclusions

Hybridizing synthetic lipid nanoparticles with natural EVs represents a promising strategy for developing next-generation bionanomaterials for medical applications. However, limited control over hybrid formation and lack of mechanistic insight have so far hindered progress in the field.

To address this challenge, we developed a rapid, single-step ethanol-assisted microfluidic method to generate hybrid biogenic nanoparticles by combining synthetic LCNPs with RBCEVs. While passive fusion of LCNPs and EVs typically results in the loss of the original LCNP structural arrangement, the ethanol-mediated hybridization preserves the cubic arrangement. This allows for maintaining the unique benefits of LCNPs, such as the high membrane curvature associated with enhanced fusogenicity, and increased cargo-loading capacity. Notably, the hybridization induces a cubic-Im3m to cubic-Im3m phase transition, driven by the incorporation of RBCEV-derived lipids into the cubosome matrix. Simultaneously, native RBCEV membrane proteins are efficiently encapsulated within amorphous, phase-separated domains reminiscent of lipid rafts in biological membranes. Functional assays confirmed that the RBCEV transmembrane protein acetylcholinesterase retained its enzymatic activity posthybridization. Furthermore, cellular uptake studies revealed enhanced internalization of the hybrid LCNPs in HEK293t cells, compared to their fully synthetic counterparts.

Mechanistic studies revealed the critical role of ethanol in promoting hybridization. We showed that 25% v/v ethanol during microfluidic mixing increases EV membrane permeability and deformability, while simultaneously inducing the formation of intermediate sponge-phase LCNPs. These intermediates act as transient states that facilitate RBCEV integration without compromising the internal LCNP structure.

Together, these findings provide a scalable and high-throughput platform for producing hybrid biogenic LCNPs with high structural and compositional fidelity. Beyond enabling efficient incorporation of biological components, our ethanol-assisted strategy ensures preservation of the original LCNP architecture, supporting both the functional integrity and the high loading capacity of synthetic nanocarriers.

Our approach is broadly applicable to a wide range of lipid-based nanoparticle systems (including conventional LNPs for NA delivery) and opens new opportunities for engineering next-generation gene delivery platforms with improved biocompatibility, nonimmunogenicity, and tissue-specific targeting.

## Supplementary Material



## Notes

Red blood cell-derived extracellular vesicle production and separation; microfluidic chip manufacturing; ethanol content quantification via refractive index measurements; assessment of vesicle membrane rigidity via nanoplasmonic assay; supplementary SAXS and DLS data, together with data analysis; supplementary cryo-EM images and cryo-EM image analysis; supplementary fluorescence measurements; SAXS data for hybrid formation in the presence of 25% (v/v) ethanol, performed without the microfluidic device; refractive index values used to evaluate ethanol concentration in LCNP preparations (PDF)
