# Supplementary material for: Ethanol-Guided Hybridization of Extracellular Vesicles with Liquid-Crystalline Lipid Nanoparticles
Source: ACS Appl Mater Interfaces. 2026 Jan 26;18(4):6530–48. doi: 10.1021/acsami.5c22972 (PMC12884453; doi:10.1021/acsami.5c22972)
Supplement: Supplementary file 1 [file am5c22972_si_001.pdf]

Supporting Information

Ethanol-Guided Hybridization of Extracellular Vesicles with Liquid-Crystalline Lipid Nanoparticles

Valentina Pacciani[1,2], Jacopo Cardellini[1,2], Arianna Balestri[1,2], Marta Rojas-Rodríguez[3], Martino Calamai[3,4], Mattia Tiboni[5], Luca Casettari[5], Catherine E Saunders[6], Anam A Karimi[6], Gennaro Sanità[7], Emanuela Esposito[7], Andrea Zendrini[2,8], Annalisa Radeghieri[2,8], Lucia Paolini[2,9], Paolo Bergese[2,8], Costanza Montis[1,2], Lucrezia Caselli[1,2*], and Debora Berti[1,2*].

Affiliations:

[1]Department of Chemistry "Ugo Schiff", University of Florence, 50019 Sesto Fiorentino, Florence, Italy

[2]CSGI, Center for Colloid and Surface Science, Sesto Fiorentino 50019 Florence, Italy

[3]European Laboratory for Non-Linear Spectroscopy, via Nello Carrara 1, 50019 Sesto Fiorentino, Italy

[4]National Institute of Optics-National Research Council (CNR-INO), 50125 Sesto Fiorentino, Italy

[5]Department of Biomolecular Sciences, University of Urbino Carlo Bo, Via Ca' le Suore, 2, 61029 Urbino, Italy

[6]SPARTA Biodiscovery, Scale Space White City, 58 Wood Lane London, W12 7RZ, United Kingdom

[7]EYE-Lab - Institute of Applied Sciences and Intelligent Systems - National Research Council, Via Pietro Castellino 111, 80131 Napoli, Italy

[8]Department of Molecular and Translational Medicine, University of Brescia, 25123, Brescia, Italy.

[9]Department of Medical and Surgical Specialties, Radiological Sciences and Public Health, University of Brescia, 25123, Brescia, Italy.

*Corresponding authors:
lucrezia.caselli@unifi.it, debora.berti@unifi.it

S1. Supplementary Methods

S1.1 Red Blood Cell-derived Extracellular Vesicle production and separation. RBC-derived extracellular vesicles (RBC-EVs) were generated through chemical induction using $CaCl_2$ and a calcium ionophore, following a previously established approach[1]. In summary, 300 mL of RBC concentrates underwent centrifugation at 1,000 × g for 8 minutes at 4°C, followed by two washes with sterile phosphate-buffered saline (PBS) devoid of calcium (Ca^{2+}) and magnesium (Mg^{2+}). The resulting RBC pellet was resuspended and washed with 50 mL of sterile PBS supplemented with 0.1 g/L $CaCl_2$ (hereafter referred to as CPBS) at a 2:1 CPBS-to-RBC volume ratio, and centrifuged again at 1,000 × g for 8 minutes at 4°C. The washed RBCs were then resuspended in 30 mL of fresh CPBS containing 10 μM calcium ionophore, transferred to 175 cm^2 tissue culture flasks (d), and incubated

for 15 hours at 37°C with 5% CO_2. Following incubation, RBCs were diluted at 1:1 in PBS, and RBC-EVs were isolated through a series of sequential centrifugation steps, all performed at 4°C: 600 × g for 20 minutes, 1,600 × g for 15 minutes, 3,200 × g for 15 minutes, and 10,000 × g for 30 minutes. At each stage, supernatants were collected while pellets were discarded. The final supernatant from the 10,000 × g step was filtered using 0.45 µm PES syringe filters and subsequently ultracentrifuged at 50,000 × g for 70 minutes at 4°C. The RBC-EV pellet was carefully resuspended in 2 mL of PBS and layered onto 2 mL of ice-cold PBS resting above a frozen 1 mL sucrose cushion (60% sucrose in PBS). Samples were ultracentrifuged at 50,000 × g for 16 hours at 4°C with the deceleration set to 0. The resulting RBC-EV-enriched red layers were collected, resuspended in 1.5 mL of PBS, and washed via ultracentrifugation at 50,000 × g for 70 minutes at 4°C. Finally, RBC-EVs were resuspended in 1 mL of PBS, diluted tenfold, aliquoted, characterized, and stored at –80°C until further analysis.

All centrifugation steps below 10,000 × g were conducted using a Eppendorf 5804R centrifuge with an A-4-44 swinging bucket rotor and 50 mL sterile disposable Falcon tubes. The 10,000 × g step was performed on a Beckman Avanti J-25 with a JA-20 fixed-angle rotor using 40 mL polypropylene screw-cap tubes. The first ultracentrifugation step was conducted on an Optima XPN-100 ultracentrifuge equipped with a TY45 Ti fixed-angle rotor and 50 mL polycarbonate tubes. The sucrose cushion was carried out using a benchtop Beckman Optima MAX-XP ultracentrifuge with an MLS-50 swinging bucket rotor in 7 mL polypropylene round-bottom tubes. The final washing was performed using the same benchtop ultracentrifuge, fitted with a TLA-55 fixed-angle rotor and 1.5 mL disposable polypropylene ultracentrifuge tubes.

All procedures involving RBCs and RBC-EV handling, including resuspension, washing, and transfer, were conducted under sterile conditions. Non-sterile disposable tubes were sterilized via autoclaving, while reusable tubes were decontaminated using a 10% H_2O_2 solution, followed by multiple rinses with Milli-Q water, as per the manufacturer's guidelines. Metal components, such as MLS-50 rotor buckets and ultracentrifuge tube caps, were sanitized with 70% ethanol, rinsed with Milli-Q water, and dried overnight under UV-light in sterile conditions.

RBCs were obtained from anonymized healthy volunteers under written consent and provided by the ASST Spedali Civili di Brescia, ethical approval "EritrEV NP5705.

S1.2 Microfluidic chip manufacturing

The microfluidic chips were produced using a Fused deposition modeling (FDM) Ultimaker 3 3D printer (Ultimaker, The Netherlands) as reported before[2]. Briefly, STL files were printed at 25 mm/s and 220 °C through a 0.25 mm nozzle, with an infill density of 100 %, a build plate temperature of 85 °C, and a polypropylene adhesion sheet using a polypropylene filament. A layer height of 0.1 mm was employed, printed in a pattern perpendicular to the device length, ensuring leak-free, semi-transparent channels. Stainless steel probe needles facilitated chip-to-pump connections by PTFE tubing.

S1.3 Ethanol content quantification via refractive index measurements

Ethanol content during dialysis was quantified using an Abbe refractometer (Atago™ Abbe NAR-1T Liquid Refractometer). A calibration curve was obtained using ethanol/water mixtures ranging from 0 to 30% v/v. To replicate the ethanol removal process occurring during dialysis of LCNPs dispersions, a 25% v/v ethanol solution in water was dialyzed under the same conditions, and aliquots were collected at defined time points. The refractive index (RI) of each sample was measured and converted to ethanol concentration using the calibration curve. These values are reported in Table S3. Based on these results, the appropriate time points for sampling LCNPs at target ethanol concentrations (25%, 20%, 15%, 10%, and 0%) were calculated.

S1.4 Assessment of Vesicle Membrane Rigidity via Nanoplasmonic Assay

Anionic gold nanoparticles (AuNPs) with an average diameter of ~12 nm were synthesized following the Turkevich–Frens method[3]. Briefly, 20 mL of 1 mM $HAuCl_4$ aqueous solution was brought to boiling under vigorous magnetic stirring, followed by the rapid addition of 2 mL of 1% (w/v) sodium citrate solution. The mixture was maintained at boiling temperature for 10 minutes. The suspension was then slowly cooled to room temperature.

To probe vesicle membrane stiffness, a nanoplasmonic assay based on AuNP clustering was performed. For each condition, 20 µL of AVs (4 mg/mL in water) were mixed with 20 µL of a water/ethanol solution to achieve the desired ethanol concentration. Then, 300 µL of the as-prepared AuNP dispersion (6 nM) was added to each mixture and allowed to interact for 5 minutes at room temperature. Finally, 700 µL of ultrapure water was added. UV–Vis spectra were then collected using a Cary 3500 UV–Vis spectrophotometer. Each condition was measured in triplicate and averaged.

To quantify vesicle-induced AuNP clustering, an aggregation index (A.I.) was calculated by dividing the area under the absorbance curve in the 560–800 nm region by the total area from 350–800 nm, as previously reported. The resulting values were normalized to the A.I. of the neat AuNP suspension. A value of 1 corresponds to non-interacting, dispersed AuNPs. This A.I. reflects vesicle membrane rigidity: increased clustering (higher A.I.) is associated with softer membranes.[4]

S2. Supplementary Figures

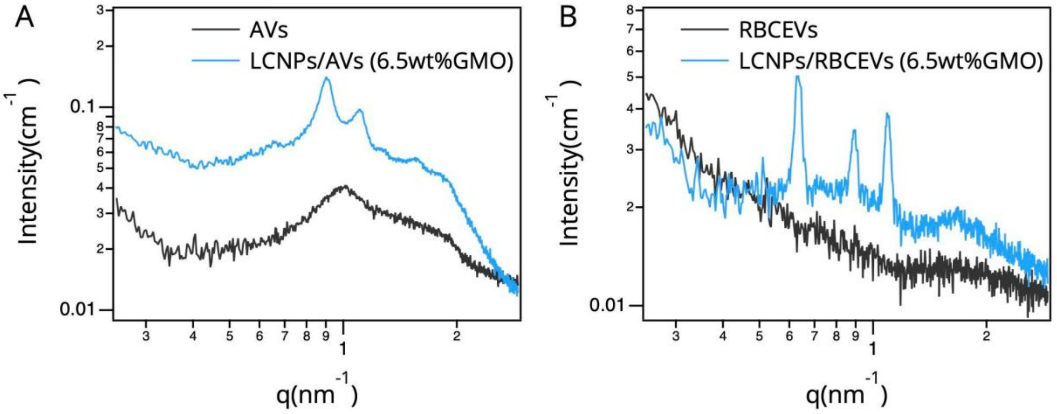

Figure S1. SAXS profiles comparing (A) AVs with LCNP/AVs hybrids and (B) RBCEVs with LCNP/RBCEVs hybrids. The concentration of AVs or RBCEVs is 1.3 mg/mL (black curves) and corresponds to the quantity present in the respective hybrid formulations.

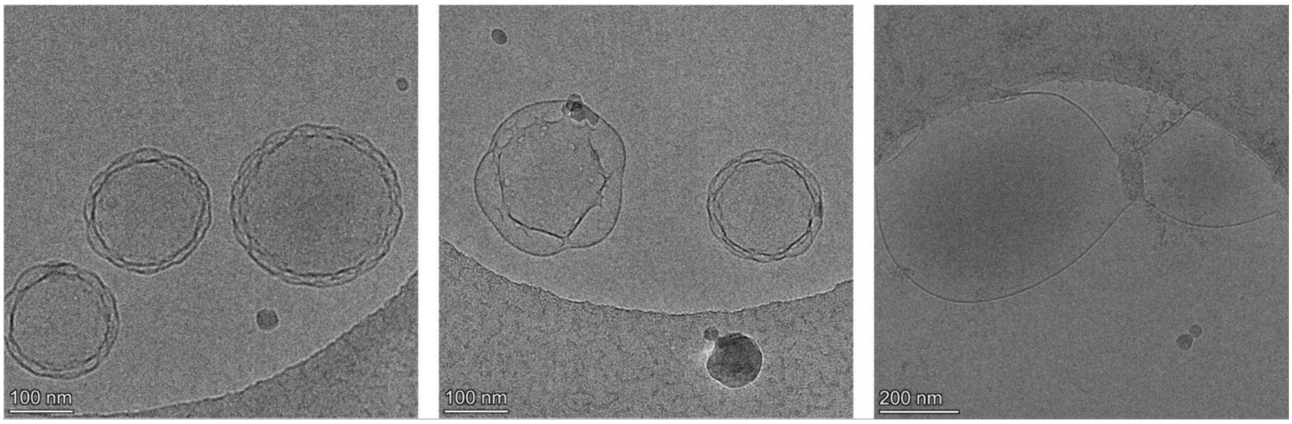

Figure S2. Additional Cryo-EM images of LCNPs mixed with AVs after 24 hours of interaction.

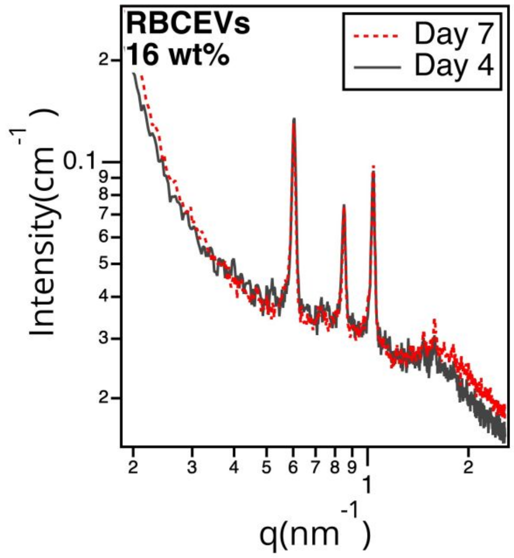

Figure S3. SAXS profiles of LCNPs/RBCEVs hybrids over time. The black curve shows the scattering profile measured after 4 days of incubation, while the red dashed curve represents the profile after 7 days.

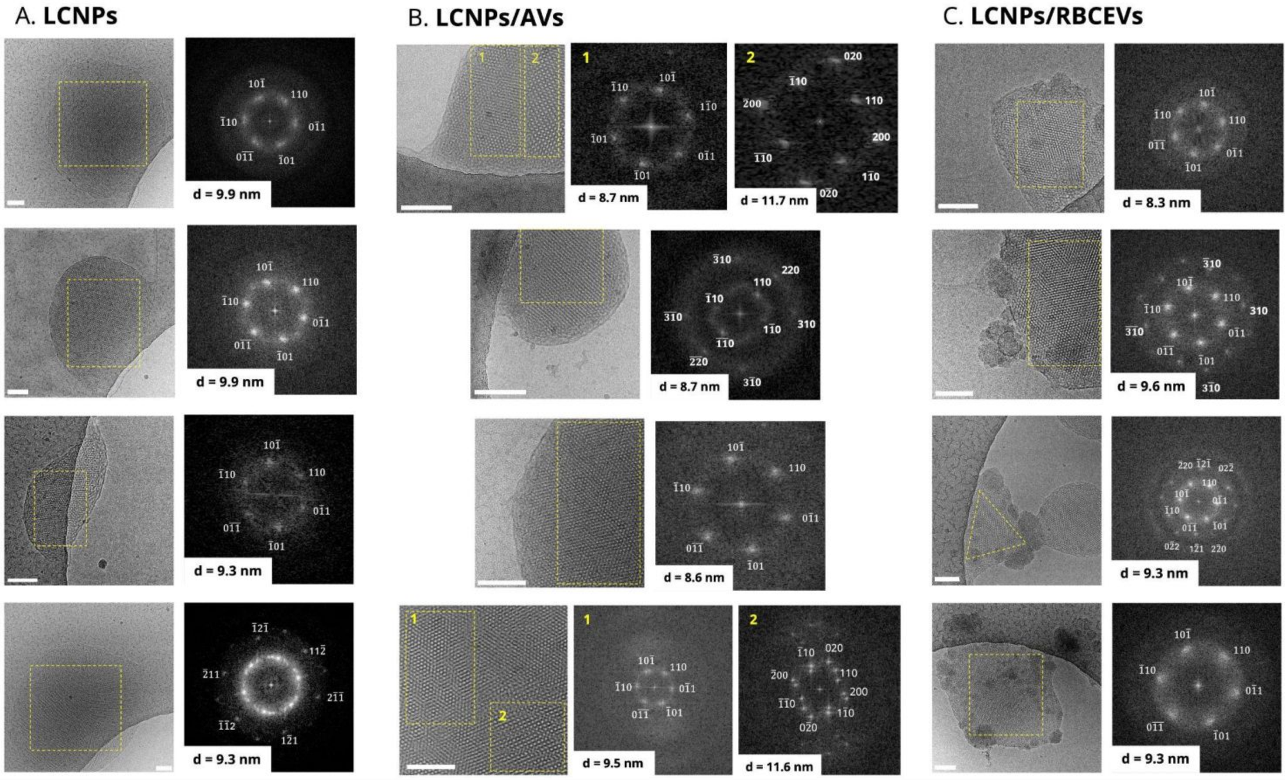

Figure S4. Cryo-EM images and corresponding Fast Fourier Transforms (FFTs) of (A) neat LCNPs, (B) LCNPs/AVs, and (C) LCNPs/RBCEVs. FFT analysis was applied to selected internal regions of cubosomes (highlighted by yellow boxes) using ImageJ. Spatial frequencies of each diffraction spot were measured, corresponding to the reciprocal of real-space interplanar spacings (*d*□□□). Miller indices (hkl) were assigned based on the symmetry of the cubic phases identified by SAXS (*Pn*3*m* or *Im*3*m*). The cubic lattice parameter *d* was calculated for each spot using the equation: *a = 1/d*□□□ × √(*h² + k² + l²*). The estimated lattice parameter (*d*) is reported as an inset in each FFT panel. Scale bar = 100 nm.

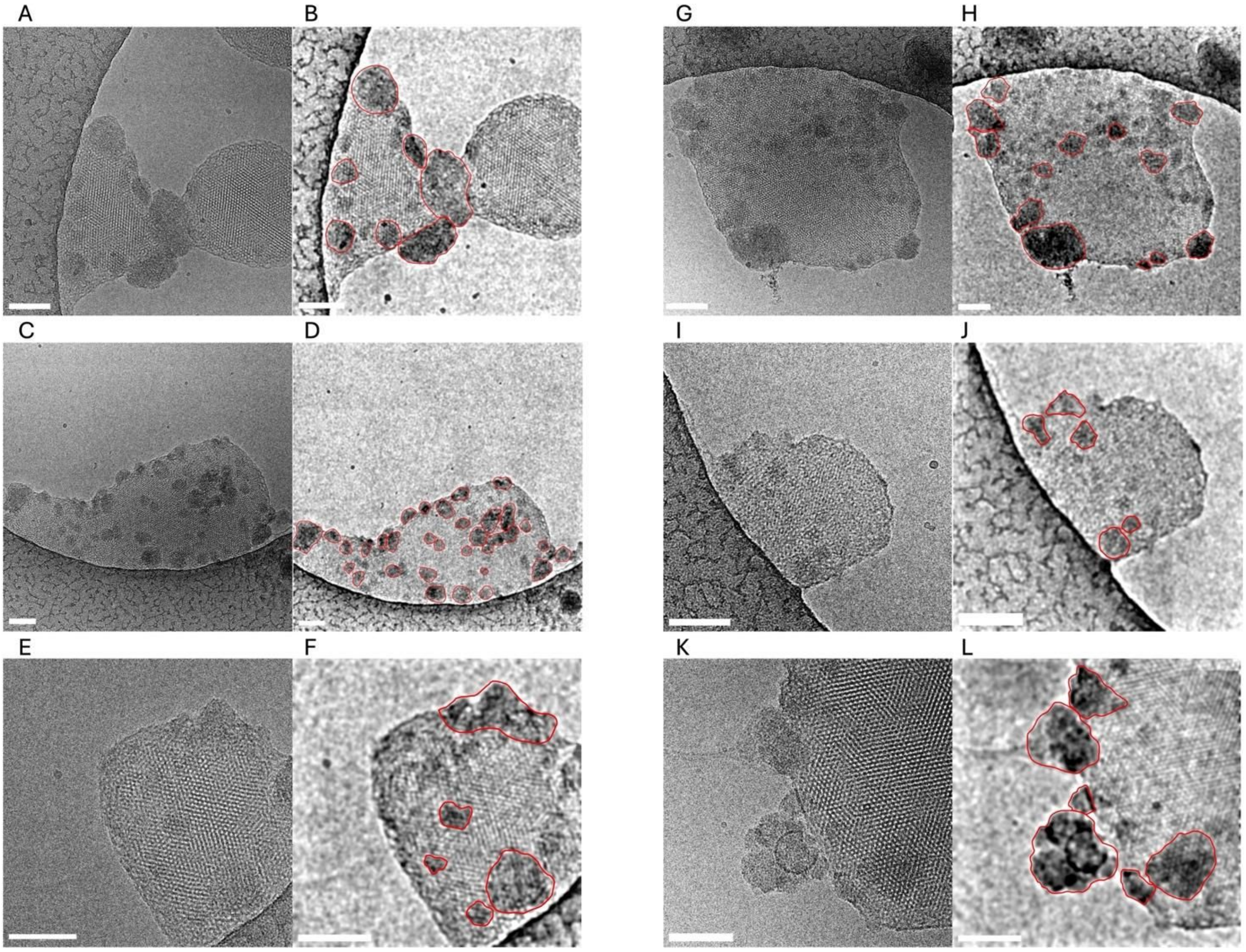

Figure S5. (A, C, E, G, I, K) Representative Cryo-EM images of LCNP/RBCEV hybrids displaying phase-separated electron-dense domains. (B, D, F, H, I, L) Corresponding images after applying a bandpass filter in ImageJ to enhance the contrast of the dark phase-segregated areas. The contours of these regions, indicated by red outlines, have been manually traced. The average area, diameter, and circularity of these domains were subsequently quantified using ImageJ's built-in functions. Scale bars 100 nm.

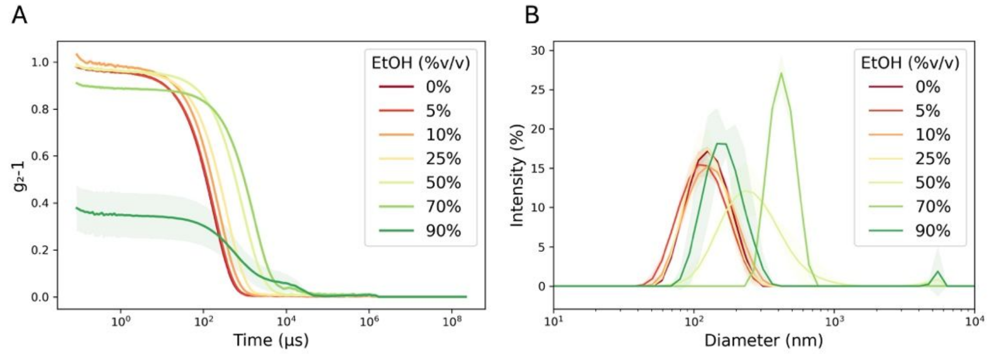

C

EtOH (v/v%)	Dh (nm)	PDI
**0**	121 ± 3.1	0.09 ± 0.01
**5**	116 ± 2.6	0.10 ± 0.01
**10**	116 ± 2.0	0.13 ± 0.02
**25**	120 ± 1.3	0.12 ± 0.02
**50**	232 ± 3.7	0.26 ± 0.01
**70**	461 ± 25.8	0.19 ± 0.03
**90**	301 ± 29.2	0.75 ± 0.16

Figure S6. (A) DLS autocorrelation functions and (B) intensity-weighted size distributions of AVs at increasing ethanol concentrations, derived using the non-negative least squares (NNLS) algorithm. (C) Summary table of hydrodynamic diameter (Dh) and polydispersity index (PDI) obtained from cumulant analysis of the autocorrelation functions.

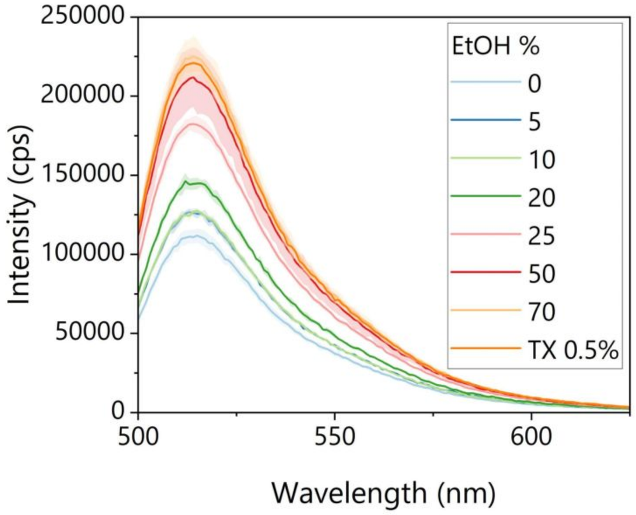

Figure S7. Fluorescence emission spectra of CF-loaded AVs after exposure to different conditions. CF-loaded AVs at a concentration of 0.5 mg/mL were incubated for 1 hour at room temperature with water/ethanol mixtures at varying ethanol concentrations or with 0.5% v/v Triton X-100 (TX). Following incubation, all samples were diluted 1:200 in Tris buffer (pH 7.4) before fluorescence measurement. The signal at 0% ethanol represents the residual fluorescence of intact vesicles (baseline), while the TX-treated sample was used as the reference for 100% dye release. All measurements were performed in triplicate and averaged.

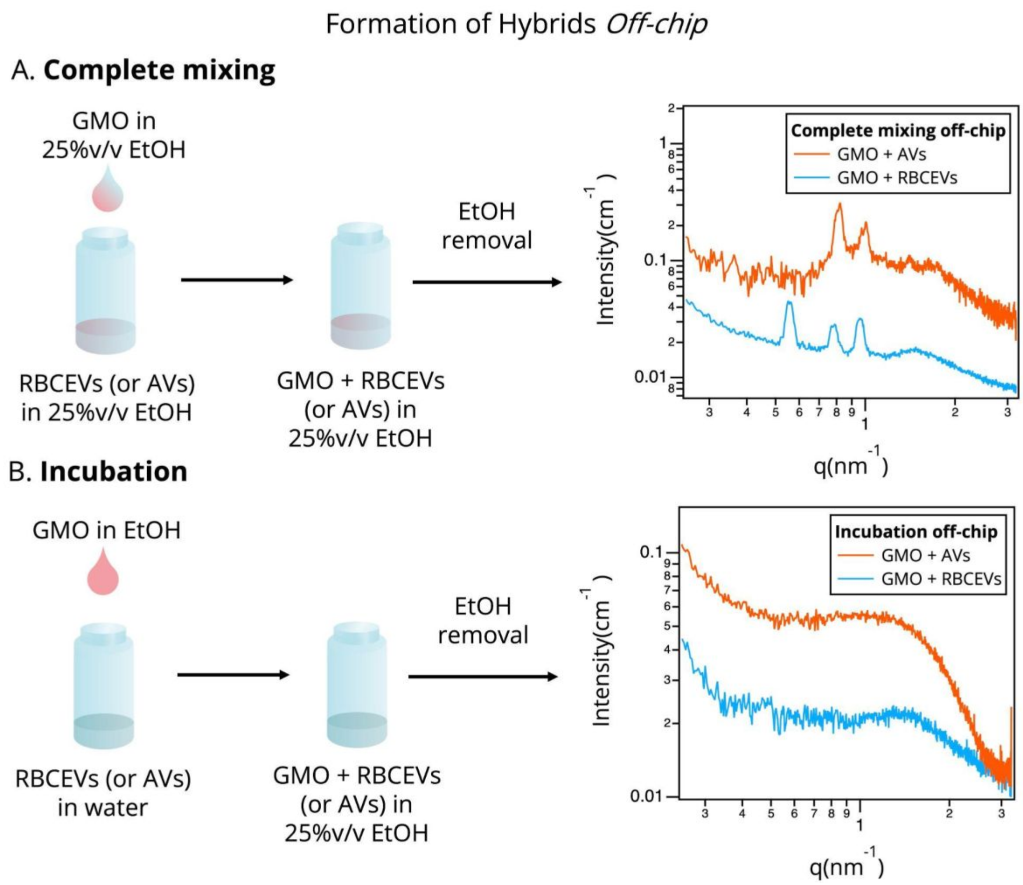

Figure S8. Schematic representation and SAXS profiles of: (A) off-chip incubation of GMO in ethanol (EtOH) with RBCEVs or AVs in water, yielding a final ethanol concentration of 25% v/v. In the absence of efficient solvent mixing, contact between components is limited, preventing hybrid formation. (B) Complete mixing of GMO and vesicles, both prepared in 25% v/v EtOH, resulting in the formation of liquid crystalline nanoparticles. In both cases, vesicles (RBCEVs or AVs) were added at 6.5 wt% relative to GMO.

S3. Supplementary Tables

Table S1. Lattice parameters and radius of water channel for LCNPs, LCNP/AVs, and LCNP/RBCEVs at varying ethanol concentrations (% v/v), derived from SAXS measurements reported in Figure 7. For samples in the sponge (L3) phase, the lattice parameter *d* was estimated from the position of the broad correlation peak using $a = 2\pi/q$. For cubic phases, a was calculated from Bragg reflections using $a = 2\pi/q_{hkl}\sqrt{(h^2 + k^2 + l^2)}$, where q_{hkl} corresponds to the position of the indexed (hkl) peak.

Sample	EtOH (v/v%)	Phase	d (nm)	R_w (nm)
LCNPs	25	L3	14.9	2.4
	20	Pn3m	12.4	2.0
	15	Pn3m	11.3	1.6
	10	Pn3m	10.9	1.5
	0	Pn3m	10.6	1.4
LCNPs/AVs	25	L3	18.7	3.1
	20	Pn3m	12.8	2.1
	15	Pn3m	12.4	2.0
	10	Pn3m	12.9	2.2
	0	Pn3m	11.3	2.1
LCNPs/RBCEVs	25	L3	22.8	6.1
	20	L3	12.0	3.2
	15	Im3m / Pn3m	14.8 / 11.9	4.0 / 1.8
	10	Im3m / Pn3m	15.5 / 12.2	4.3 / 1.9
	0	Im3m	13.7	3.6

Table S2. Refractive index values and corresponding ethanol concentrations (% v/v) measured at different time points during dialysis of a 25% v/v ethanol solution.

Time (min)	R.I.	% EtOH
0	1.346	25
13	1.342	15
35	1.339	8
50	1.338	5
80	1.337	3
110	1.336	1
150	1.335	0

References

(1) Usman, W. M.; Pham, T. C.; Kwok, Y. Y.; Vu, L. T.; Ma, V.; Peng, B.; Chan, Y. S.; Wei, L.; Chin, S. M.; Azad, A.; He, A. B. L.; Leung, A. Y. H.; Yang, M.; Shyh-Chang, N.; Cho, W. C.; Shi, J.; Le, M. T. N. Efficient RNA Drug Delivery Using Red Blood Cell Extracellular Vesicles. *Nat Commun* 2018, *9* (1). https://doi.org/10.1038/s41467-018-04791-8.

(2) Tiboni, M.; Tiboni, M.; Pierro, A.; Del Papa, M.; Sparaventi, S.; Cespi, M.; Casettari, L. Microfluidics for Nanomedicines Manufacturing: An Affordable and Low-Cost 3D Printing Approach. *Int J Pharm* 2021, *599*. https://doi.org/10.1016/j.ijpharm.2021.120464.

(3) Turkevich, J.; Stevenson, P. C.; Hillier, J. A Study of the Nucleation and Growth Processes in the Synthesis of Colloidal Gold. *Discussions of the Faraday Society*. 1951. https://doi.org/10.1039/DF9511100055.

(4) Caselli, L.; Ridolfi, A.; Cardellini, J.; Sharpnack, L.; Paolini, L.; Brucale, M.; Valle, F.; Montis, C.; Bergese, P.; Berti, D. A Plasmon-Based Nanoruler to Probe the Mechanical Properties of Synthetic and Biogenic Nanosized Lipid Vesicles. *Nanoscale Horiz* 2021, *6* (7). https://doi.org/10.1039/d1nh00012h.
